# Supplementary material for: Examining risk factors for self-harm and suicide in LGBTQ+ young people: a systematic review protocol
Source: BMJ Open. 2019 Nov 11;9(11):e031541. doi: 10.1136/bmjopen-2019-031541 (PMC6858144; doi:10.1136/bmjopen-2019-031541)
Supplement: Supplementary data [file bmjopen-2019-031541supp002.pdf]

SUPPLEMENTARY FILE 2

Search strategy terms:

(self-harm OR self harm* OR self-injur* OR "self injur*" OR self-cut* OR self-destruct* OR “self destruct*” OR "nonsuicidal self-injur*" OR "non-suicidal self injur*" OR "deliberate self harm" OR “deliberate self-harm” OR DSH OR "self-mutil*" OR overdos* OR self-inflicted injur* OR "self inflicted injur*" OR suicid* OR “parasuicid*” OR para-suicid* OR parasuicid* OR suicidal behav* OR suicide* OR "life-threatening behavio*" OR “suicide ideat*” OR “suicide attempt*” OR “attempted suicide*” OR NSSI)

AND

(moderat* OR mediat* OR "risk facto*" OR mechan* OR predict* OR pathway OR interact* OR "protective facto*" OR facto* OR influence OR correlate* OR precurs* OR “causal facto*”)

AND

(transgender* OR transsexual* OR "gender nonconforming" OR "gender identity disorder" OR "gender dysphoria" OR "gender minority" OR lesbian*OR gay* OR bisexual* OR "sexual minority" OR "same-sex" OR homosexual* OR “homosexuality, male” OR “homosexuality, female” OR “gender identity” OR non-heterosexual* OR “non heterosexual*”OR homosexuality OR queer* OR questioning OR "non-binary" OR "non binary" OR “LGBT*” OR “sexual dissident*” OR “sexual and gender minorities” OR “gender variant” OR gender-variant OR genderqueer OR intersex OR “minority groups” OR “TGNC” OR “transgender and gender nonconforming”)

AND

(Child* OR adolesc* OR "young people" OR kid* OR pupils OR youth OR juvenile OR “young adult*” OR “young person” OR minor*)
