# Supplementary material for: Examining risk factors for self-harm and suicide in LGBTQ+ young people: a systematic review protocol
Source: BMJ Open. 2019 Nov 11;9(11):e031541. doi: 10.1136/bmjopen-2019-031541 (PMC6858144; doi:10.1136/bmjopen-2019-031541)
Supplement: Author's manuscript [file bmjopen-2019-031541.draft_revisions.pdf]

BMJ Open is committed to open peer review. As part of this commitment we make the peer review history of every article we publish publicly available.

When an article is published we post the peer reviewers' comments and the authors' responses online. We also post the versions of the paper that were used during peer review. These are the versions that the peer review comments apply to.

The versions of the paper that follow are the versions that were submitted during the peer review process. They are not the versions of record or the final published versions. They should not be cited or distributed as the published version of this manuscript.

BMJ Open is an open access journal and the full, final, typeset and author-corrected version of record of the manuscript is available on our site with no access controls, subscription charges or pay-per-view fees (http://bmjopen.bmj.com).

If you have any questions on BMJ Open's open peer review process please email info.bmjopen@bmj.com

BMJ Open

A systematic review examining risk factors for self-harm and suicide in LGBTQ+ young people

Journal:	*BMJ Open*
Manuscript ID	bmjopen-2019-031541
Article Type:	Protocol
Date Submitted by the Author:	09-May-2019
Complete List of Authors:	Williams, A. Jess; University of Birmingham, School of Psychology<br>Arcelus, J; University of Nottingham , School of Medicine<br>Townsend, Ellen; University of Nottingham, School of Psychology<br>Michail, Maria; University of Birmingham, School of Psychology
Keywords:	Suicide & self-harm < PSYCHIATRY, sexual identity, gender identity, young people

SCHOLARONE™
Manuscripts

Systematic Review Protocol [Version 6, May 2019]

A systematic review examining risk factors for self-harm and suicide in LGBTQ+ young people

A. Jess Williams*[1,2] (a.williams.10@pgr.bham.ac.uk), Jon Arcelus[3] (jon.arcelus@nottingham.ac.uk), Ellen Townsend[2] (ellen.townsend@nottingham.ac.uk), Maria Michail[1] (m.michail@bham.ac.uk)

[1]Institute for Mental Health, School of Psychology, University of Birmingham, UK

[2]Self-Harm Research Group, School of Psychology, University of Nottingham, UK

[3]Institute of Mental Health, School of Medicine, University of Nottingham, UK

* Corresponding author

52 Pritchatt's Road,

School of Psychology

Institute for Mental Health

University of Birmingham

B15 2TT

a.williams.10@pgr.bham.ac.uk

Tel. +(44)121 4142855

Word count: 2264 (excluding title page, abstract, references, figures and tables)

Key words:

Self-harm, suicide, sexual identity, gender identity, young people

For peer review only

ABSTRACT

Introduction: Young people who identify as LGBTQ+ are at increased risk for self-harm, suicide ideation and behaviours. However, there has yet to be a comprehensive understanding of what risk factors influence these behaviours within LGBTQ+ young people as a whole. The purpose of this systematic review is to examine risk factors associated with self-harm, suicidal ideation and behaviour in LGBTQ+ young people.

Methods and analysis: A systematic review and meta-analysis will be conducted, conforming to the reporting guidelines of the Preferred Reporting Items for Systematic Reviews and Meta-Analyses (PRISMA) statement recommendations. Electronic databases (MEDLINE, Scopus, EMBASE , PsycINFO, and Web of Science) will be systematically searched for cross-sectional, prospective, longitudinal, cohort, and case-control designs which examine risk factors for self-harm and/or suicidal ideation and behaviour in LGBTQ+ young people. Only studies published in English will be included. No date restrictions will be applied. Study quality assessment will be conducted using a modified Newcastle-Ottawa Scale. A meta-analysis will be performed if appropriate; alternatively findings will be drawn together in a narrative synthesis.

Ethics and dissemination: This is a systematic review of published literature and thereby ethical approval was not sought. The review will be submitted to a peer-reviewed journal, be publicly disseminated at conferences focusing on mental health, self-harm and suicide prevention. The findings will also be shared through public engagement and involvement, particularly those related to young LGBTQ+ individuals.

PROSPERO registration number: CRD42019130037

STRENGTHS AND LIMITATIONS OF THIS STUDY

- This is the first systematic review of risk factors for self-harm, suicidal ideation and behaviour among LGBTQ+ young people.
- The protocol has been written following the PRISMA-P guidelines (2015).
- A limitation of this systematic review is the exclusion of studies which are not published in English; this may bias findings against non-English speaking countries.

For peer review only

For peer review only - http://bmjopen.bmj.com/site/about/guidelines.xhtml

INTRODUCTION

Suicide, intentionally ending one's own life[1], is one of the leading causes of death within young people[2-3]. Globally, suicide accounts for around 131,441 deaths between the ages of 15-24[2]. Rates of self-harm, the self-injury or poisoning of one's self irrespective of suicidal intent[4], are on the rise among young people. Data from the 2014 Adult Morbidity Survey states that 25.7% of women and 9.7% of men were likely to have self-harmed between the ages of 16-24 years[5]. Approximately 50% of those who die by suicide have previously self-harmed, making self-harm one of the strongest predictors of suicide[6-8].

Among people who identify under the umbrella term LGBTQ+ (Lesbian, Gay, Bisexual, Transgender, Queer or Questioning), this being how one self-identifies as their sex, sexuality, gender identity and gender expression[9], there are elevated rates of suicidal ideation (thoughts of self-harm, the consideration or planning of suicide[1]) and suicidal behaviour[10-12]. King et al's 2008 systematic review found that lesbian, gay and bisexual (LGB) individuals were found to be at higher risk for both suicidal behaviour and ideation compared to heterosexuals[10]. Internationally, this has been estimated to be between four to eight times higher in LGB and transgender (T) young people when compared to the heterosexual and cisgender peers[13]. While as a broad group, sexual and gender minorities have high levels of suicidal ideation and behaviour, among T people (individuals who do not present or identify with their sex assigned at birth[14]) these rates are even greater. Indeed, lifetime risk for suicide attempt is estimated to be between 22-43%, with 9-10% having made an attempt in the past 12 months[15-19].

Previous research has examined potential risk factors for self-harm and suicide in young people in general populations. These risk factors range from demographic characteristics such as being female, being younger or having fewer qualifications[20], to childhood abuse and neglect[21-22]. Additional risks such as bullying and academic pressure have been linked to suicidal behaviour in young people[23]. Other factors which relate to the risk of self-harm repetition include troubled relationships with family members, social isolation, poor academic performance, alcohol and drug misuse, and depression[24].

Given the heightened risk of suicide attempt within LGBTQ+[10-12, 25], it would be pertinent to explore which factors specifically pose as a risk to this population. Although some of these risk factors may overlap with those from non-LGBTQ+ populations (e.g. depression, substance or alcohol misuse)[10, 26], there are also unique risk factors to sexual and gender minorities. For example, Clements-Nolle et al[26] stated that experiences of abuse, discrimination or harassment due to an individual's gender identity or presentation were also linked to high levels of attempted suicide. Meyer[27-28] suggested that additional risk within homosexual communities may be related to the high levels of stigma, prejudice and discrimination which thereby impacts individual's mental wellbeing. Victimisation or discrimination due to sexual orientation and gender identity is particularly common within LGBTQ+ populations[29-32]. In addition, among LGBTQ+ young people these experiences were found to be significantly associated with suicidal ideation[33-34]. It is suggested that young people may internalise these experiences of public stigma in relation to being LGBTQ+, which can lead to adverse impacts on their self-perception and beliefs, which could then enhance suicidal ideation, behaviour or self-harm[35].

For peer review only - http://bmjopen.bmj.com/site/about/guidelines.xhtml

Why this review is important

The goal of this systematic review is to comprehensively examine risk factors for self-harm and suicide ideation and behaviour within LGBTQ+ young people. Identifying and understanding how these factors relate to self-harm and suicide could allow future research to address specific risks and streamline potential studies to target the mental health needs of this population. Previous reviews regarding various dimensions of self-harm[21-22, 36-38] have focused on a specific subgroup, such as the prevalence within transgender population[39]. Whereas, few have considered both self-harm and suicide in relation to LGBTQ+ populations as a broad group[10, 40-41]. Thus, this review not only takes a dimensional approach to include self-harmful thoughts and behaviour with and without intent of suicide, thereby covering a wide spectrum of suicide and self-harm, but also includes all sexual and gender minorities.

Objective: To examine risk factors associated with self-harm and suicide ideation and behaviour in LGBTQ+ young people.

METHODS

A systematic review of empirical quantitative studies which examine risk factors for self-harm and suicidal ideation and behaviour in LGBTQ+ young people will be conducted. The search will consider all studies up to the 1st of April 2019. This protocol follows the Preferred Reporting Items for Systematic Reviews and Meta-Analyses Protocol (PRISMA-P) guidelines[42], presented in the supplementary file 1. Study quality will be assessed by the Newcastle-Ottawa Scale (NOS)[43] and the adapted version for cross-sectional studies[44]. The protocol for this article has been registered with PROSPERO: CRD42019130037.

Eligibility criteria: see table 1 for summary:

Types of studies: This will include empirical quantitative peer reviewed studies following cross-sectional, prospective, longitudinal, cohort, and case-control designs which examine risk factors for self-harm and/or suicidal ideation and behaviour in LGBTQ+ young people. These papers may specifically focus on one subgroup of the LGBTQ+ umbrella (e.g. transgender) or look across groups (e.g. LGB). Mixed-method papers which have applicable extractable information will also be included. Grey literature such as theses, dissertations or conference proceedings will not be included. Commentary, reviews, editorial or opinion pieces will also be excluded. All included studies must be available as full-text and peer-reviewed in the English language.

Types of participants: Participants between the ages of 12-25 years old, who identify as LGBTQ+ (Lesbian, Gay, Bisexual, Transgender, Queer or Questioning) referring to how one self-identifies as their sex, sexuality, gender identity and gender expression[9]. Participants will also have had experiences of self-harm (self-injury or poisoning irrespective of suicidal intention[4], suicide ideation (thoughts of self-harm, the consideration or planning of a suicidal attempt e.g. method[1]or suicidal behaviour (intentionally trying to end one's own life[1]. Alternatively, if participants met the above inclusion criteria as a subgroup, this data will be extracted.

For peer review only - http://bmjopen.bmj.com/site/about/guidelines.xhtml

Types of outcome measures: Studies will be included if they used a measure for self-harm, suicidal ideation or suicidal behaviour. Consideration of the number of events (self-harm and suicidal attempts, suicidal ideation will be taken as present or not present) as described by study authors, and any standardised rating scale for assessing the presence of self-harm and suicidal ideation and behaviour. This can be specific tools for considering suicide e.g. Beck Scale for Suicide Ideation (SSI)[45]; Motto's Risk Estimator for Suicide[46]; Positive and Negative Suicide Ideation (PANSI)[47]; Columbia-Suicide Severity Rating Scale[48]; or self-harm e.g. Self-Harm Inventory (SHI)[49]; Inventory of Statements about Self-injury (ISAS)[36]; Self-Injurious Thoughts and Behaviour Interview (SITBI) Short Form[50]. Also included will be self-harm or suicide items from general scales such as items 102-105 from the Mental Health History Form[51] or individual questions regarding self-harm or suicide had ever been considered or acted upon[52] and clinician reports.

Table 1: Inclusion and exclusion criteria for papers

**Inclusion Criteria**	**Exclusion criteria**
- Peer reviewed studies.<br>- Any geographical location.<br>- English language.<br>- Empirical quantitative studies, following cross-sectional, prospective, longitudinal, cohort and case-control designs.<br>- Studies must consider factors associated with or predictive of self-harm, suicidal ideation or suicidal behaviour.<br>- Participants must be young people (12-25 years).<br>- Participants that are identified or self-identified as any sexual or gender minority or member of LGBTQ+.<br>- Participants that have had an experience of self-harm, suicidal ideation or behaviour.	- Non-peer reviewed literature.<br>- Not English language.<br>- Grey literature such as theses, dissertation or conference proceedings.<br>- Articles such as commentaries, reviews, editorial or opinion pieces.<br>- Empirical qualitative studies.<br>- Studies not considering risk factors.<br>- Sample not aged between 12 and 25 years, e.g. adults 26 years and above or children 12 years and under.<br>- Participants who are identified as heterosexual or not part of sexual or gender minority.<br>- Participants who have no experience of self-harm, suicidal ideation or suicidal behaviour.

For peer review only

Search strategy:

The search will be limited to English language and run up to 31st March 2019. No date restrictions will be applied. The following electronic bibliographic databases were searched: MEDLINE, Scopus, EMBASE, PsycINFO, and Web of Science for peer-reviewed publications which examine risk factors for self-harm or suicide within LGBTQ+ young people. The search strategy can be found in the supplementary file 2. This was developed in collaboration with an academic skill specialist from the University of Birmingham library to ensure the robustness of the search. Reference lists of eligible papers and conduct citation searches of key papers will be explored to identify additional reports. Before the final draft of the systematic review is completed, a second search will be conducted to allow for any additional studies to be identified.

Study records:

Selection process: The search strategy will retrieve titles and/or abstracts which will be screened by two independent researchers to identify studies which potentially meet the inclusion criteria outlined above. The full-texts of the studies will be retrieved and independently assessed for eligibility. Any disagreement or uncertainty over the eligibility of particular studies will be resolved through discussion.

Data management and collection: Rayyan QCRI (https://rayyan.qcri.org/welcome), the online systematic review tool, will be used to manage and screen all retrieved papers. AJW will be the solo team member responsible for adding or amending paper records in Rayyan, as well as identifying and removing duplicates. A second independent reviewer will be given access to all titles and abstracts, within Rayyan. They will be able to make their own decision as to whether to include or exclude a paper, blind to AJW's decisions. AJW will also be blind to the decisions of the second reviewer until all titles and abstracts are reviewed. Rayyan will provide information on the original number of titles screened, duplications, those excluded at this stage, and those included titles at this stage.

Those papers which reach full-text screening will be managed within Zotero (https://www.zotero.org/), the bibliographic software. These will be sectioned by those included at full-text screening, and final inclusion. Reason for exclusion will be reported for each paper. AJW will be responsible for liaising with inter-library loans and obtaining the full-text papers. Full-text screening will again be conducted by two researchers, blind to each other's decisions, who will resolve disagreements through discussion. If an agreement cannot be reached regarding a paper, this will be rated by a third researcher.

A pre-piloted standardised data extraction tool will be adapted and used on included studies[53]. Extracted information will include: 1) author and publication date; 2) study design and setting; 3) characteristics of participants (age, gender identity), and the studies' inclusion and exclusion criteria; 4) method of harm (self-harm, suicidal behaviour or ideation); 5) factors associated with or predictive of self-harm or suicidal ideation and behaviour (including clinician diagnosis, subscales or validated scales used to assess these items); 6) information relating to risk of bias for the individual study.

Risk of bias (quality assessment): The overall quality of each study will be evaluated by two independent researchers using the Newcastle-Ottawa Scale (NOS)[43] and the Newcastle-Ottawa Scale adapted for cross-sectional studies[44].

Data synthesis and assessment of heterogeneity: Descriptive results of each included study will be presented in a table format, such as details regarding study design, participants, self-harm or suicidal ideation and behaviour methods and measures, identified factors and tools used to assess these.

A meta-analysis of risk factors for self-harm and suicidal ideation and behaviour using RevMan 5 (https://community.cochrane.org/help/tools-and-software/revman-5/revman-5-download) will be conducted by pooling data on risk factors using a random-effects model with the assumption that populations have various effect sizes and weights studies from in- and between-study variance. This will consider the extent or heterogeneity, which will be determined using the I^2 statistic. From this, a summary effect will be presented, associated 95% CI and *p* values. Publication bias will be assessed using funnel plots and Harbord-Egger test[54].

For peer review only

For peer review only - http://bmjopen.bmj.com/site/about/guidelines.xhtml

Additionally, consideration will be given to subgroup analysis if data allows, exploring heterogeneity by sexual and gender minorities; such that these studies are split by LGBQ and transgender; as even within this population it is recognised that the transgender population have high levels of suicide risk and self-harm[39, 55]. This will allow us to determine which risk factors are similar within sexual and gender minorities, and those that unique to the transgender population. Further examination of risk factors will be conducted by age difference, split by 12-18years and 19-25years.

The quality of the included studies will be considered when synthesising and analysing the findings in terms of the strength of evidence which they provide. A sensitivity analysis will be run to assess the robustness of the studies[56]. If statistical pooling is not possible, findings will follow a narrative synthesis as suggested by ERSC guidelines[57]. The analysis will take place over four steps; 1) developing a theory of which factors influence LGBTQ+ young people, 2) synthesising the findings, 3) exploring whether there are relationships within the data and what these might be, 4) assessing the robustness of the synthesis.

Patient and Public Involvement: No patients involved.

ETHICS AND DISSEMINATION

This is a systematic review of published literature and thereby ethical approval was not sought. It will be submitted to a peer-reviewed journal, be publicly disseminated at conferences focusing on mental health, self-harm and suicide prevention. Findings will also be shared through public engagement and involvement, particularly considering young LGBTQ+ individuals.

DISCUSSION

This systematic review will be the first to provide a rich, holistic account of the existing evidence of risk factors for self-harm and suicidality within a broad sample of LGBTQ+ young people. The synthesis of these findings will assess the prevalence of particular factors which impact this population, which may not be relevant to non-sexual or gender minority young people. Acknowledging the similarities and differences of risk factors between sexual orientation and gender identity groups could be used as a valuable stepping stone when considering LGBTQ+ research and promoting more applicable research aims.

The quality and strength of the evidence will be rigorously assessed, which could be used to inform future research targeting these particular risks. This could ultimately help to inform self-harm and suicidality prevention within LGBTQ+ young people. It is anticipated that the findings of this review will be of interest to the wider academic and clinical community, policymakers, young people who identify as a sexual or gender minority, those with experience of self-harm or suicide.

AUTHORS CONTRIBUTIONS

For peer review only - http://bmjopen.bmj.com/site/about/guidelines.xhtml

All authors conceptualised the study. AJW developed the search strategy and conducted the literature search. AJW and MM wrote the first draft of the manuscript. All authors reviewed, edited, and approved the final manuscript.

FUNDING STATEMENT

This work was supported by the Midlands Graduate School ESRC DTP Joint Studentship awarded to A. Jess Williams. The funding body were not involved in the development of this protocol.

COMPETING INTERESTS

None declared.

For peer review only

For peer review only - http://bmjopen.bmj.com/site/about/guidelines.xhtml

REFERENCES

1. American Psychiatric Association. Diagnostic and statistical manual of mental disorders (DSM-5®). American Psychiatric Pub; 2013 May 22.
2. World Health Organization. Suicide across the world. 2016. Available from: https://www.who.int/mental_health/prevention/suicide/suicideprevent/en/. Accessed 7 January 2019.
3. Office for National Statistics. Suicides in the United Kingdom, 2017 Registration – ONS. 2018. Available at: https://www.ons.gov.uk/releases/suicidesintheuk2017registrations. Accessed 27 Feb. 2019.
4. National Collaborating Centre for Mental Health. Self-harm: Longer Term Management (NICE Clinical Guideline 133). London: National Institute for Clinical Excellence 2011.
5. McManus S, Bebbington P, Jenkins R et al. Mental health and wellbeing in England: Adult Psychiatric Morbidity Survey 2014. Leeds: NHS Digital. 2016. Available at http://digital.nhs.uk/catalogue/PUB21748. [Accessed 27 Feb. 2019]
6. Sakinofsky I. Repetition of suicidal behaviour. In: Hawton K, and Van Heeringen K eds. The International Handbook of Suicide and Attempted Suicide. Chichester: John Wiley & Sons Ltd, 2000:385–404.
7. Hawton K, Bergen H, Cooper J, et al. Suicide following self-harm: findings from the Multicentre Study of self-harm in England, 2000–2012. Journal of Affective Disorders. 2015 April;(175):147-151.
8. Rodway C, Tham SG, Ibrahim S, et al. Suicide in children and young people in England: a consecutive case series. The Lancet Psychiatry. 2016 Aug 1;3(8):751-9.
9. Jourian TJ. Evolving nature of sexual orientation and gender identity. New Directions for Student Services. 2015 Dec;(152):11-23.
10. King M, Semlyen J, Tai SS, Killaspy H, Osborn D, Popelyuk D, Nazareth I. A systematic review of mental disorder, suicide, and deliberate self harm in lesbian, gay and bisexual people. BMC psychiatry. 2008 Dec;8(1):70.
11. Plöderl M, Wagenmakers EJ, Tremblay P, Ramsay R, Kralovec K, Fartacek C, Fartacek R. Suicide risk and sexual orientation: a critical review. Archives of Sexual Behavior. 2013 Jul 1;42(5):715-27.
12. Hottes TS, Bogaert L, Rhodes AE, et al. Lifetime prevalence of suicide attempts among sexual minority adults by study sampling strategies: a systematic review and meta-analysis. American journal of public health. 2016 May;106(5):e1-2.
13. Haas AP, Eliason M, Mays VM, Mathy RM, Cochran SD, D'Augelli AR, Silverman MM, Fisher PW, Hughes T, Rosario M, Russell ST. Suicide and suicide risk in lesbian, gay, bisexual, and transgender populations: Review and recommendations. Journal of homosexuality. 2010 Dec 30;58(1):10-51.
14. Richards C, Barker M. Sexuality and gender for mental health professionals: A practical guide. Sage; 2013 Oct 15.
15. Whittle S, Turner L, Combs R, et al. Transgender EuroStudy: Legal Survey and Focus on the Transgender Experience of Health Care. Brussels, Belgium: ILGA Europe; 2008. http://tgeu.org/sites/default/files/eurostudy.pdf. Accessed 26 March 2019
16. Bauer GR, Pyne J, Francino MC, et al. Suicidality among trans people in Ontario: implications for social work and social justice/La suicidabilité parmi les personnes trans en Ontario: implications en travail social et en justice sociale. Serv Soc. 2013;59:35–62.

For peer review only - http://bmjopen.bmj.com/site/about/guidelines.xhtml

17. Motmans J, de Biolley I, Debunne S. Being Transgender in Belgium: Mapping the Social and Legal Situation of Transgender People. Brussels: Institute for the Equality of Women and Men; 2010. http://igvmiefh. belgium.be/nl/binaries/34%20-%20Transgender_ENG_tcm336-99783.pdf. Accessed 26 March 2019
18. Grant JM, Mottet LA, Tanis J, et al. Injustice at Every Turn: A Report of the National Transgender Discrimination Survey. Washington, D.C.: National Center for Transgender Equality and National Gay and Lesbian Task Force; 2011. http://www.thetaskforce.org/downloads/reports/reports/ntds_full.pdf. Accessed 26 March 2019.
19. McNeil J, Bailey L, Ellis S, et al. Mental Health Study 2012. Edinburgh: Equality Network; 2012. http://www.scottishtrans.org/ Uploads/Resources/trans_mh_study.pdf. Accessed 26 March 2019.
20. Nock MK, Borges G, Bromet EJ, et al. Cross-national prevalence and risk factors for suicidal ideation, plans and attempts. The British Journal of Psychiatry. 2008 Feb;192(2):98-105.
21. Gratz KL. Risk factors for and functions of deliberate self-harm: An empirical and conceptual review. Clinical Psychology: Science and Practice. 2003 Jun;10(2):192-205.
22. Fliege H, Lee JR, Grimm A, et al. Risk factors and correlates of deliberate self-harm behavior: A systematic review. Journal of psychosomatic research. 2009 Jun 1;66(6):477-93.
23. Rodway C, Tham SG, Ibrahim S, et al. Suicide in children and young people in England: a consecutive case series. The Lancet Psychiatry. 2016 Aug 1;3(8):751-9.
24. Hawton K, James A. Suicide and deliberate self harm in young people. BMJ. 2005;330(7496):891–4.
25. Semlyen J, King M, Varney J, et al. Sexual orientation and symptoms of common mental disorder or low wellbeing: combined meta-analysis of 12 UK population health surveys. BMC psychiatry. 2016 Dec;16(1):67.
26. Clements-Nolle K, Marx R, Katz M. Attempted suicide among transgender persons: The influence of gender-based discrimination and victimization. Journal of homosexuality. 2006 Oct 11;51(3):53-69.
27. Meyer IH. Minority stress and mental health in gay men. Journal of health and social behavior.1995 Mar;1:38-56.
28. Meyer IH. Prejudice, social stress, and mental health in lesbian, gay, and bisexual populations: conceptual issues and research evidence. Psychological bulletin. 2003 Sep;129(5):674.
29. Cochran SD, Mays VM. Lifetime prevalence of suicide symptoms and affective disorders among men reporting same-sex sexual partners: results from NHANES III. American journal of public health. 2000 Apr;90(4):573.
30. Bontempo DE, d'Augelli AR. Effects of at-school victimization and sexual orientation on lesbian, gay, or bisexual youths' health risk behavior. Journal of Adolescent Health. 2002 May 1;30(5):364-74.
31. Grossman AH, D'augelli AR, Frank JA. Aspects of psychological resilience among transgender youth. Journal of LGBT Youth. 2011 Mar 29;8(2):103-15.
32. Brandelli Costa A, Pasley A, Machado WD, et al. The experience of sexual stigma and the increased risk of attempted suicide in young Brazilian people from low socioeconomic group. Frontiers in psychology. 2017 Feb 22;8:192.

For peer review only - http://bmjopen.bmj.com/site/about/guidelines.xhtml

33. Kelleher C. Minority stress and health: Implications for lesbian, gay, bisexual, transgender, and questioning (LGBTQ) young people. Counselling psychology quarterly. 2009 Dec 1;22(4):373-9.
34. Taliaferro LA, Muehlenkamp JJ. Nonsuicidal self-injury and suicidality among sexual minority youth: risk factors and protective connectedness factors. Academic pediatrics. 2017 Sep 1;17(7):715-22.
35. McDermott E, Hughes E, Rawlings V. The social determinants of lesbian, gay, bisexual and transgender youth suicidality in England: a mixed methods study. Journal of Public Health. 2017 Oct 17;40(3):e244-51.
36. Klonsky ED. The functions of deliberate self-injury: A review of the evidence. Clinical psychology review. 2007 Mar 1;27(2):226-39.
37. Skegg K. Self-harm. The Lancet. 2005 Oct 22;366(9495):1471-83.
38. Edmondson AJ, Brennan CA, House AO. Non-suicidal reasons for self-harm: a systematic review of self-reported accounts. Journal of Affective Disorders. 2016 Feb 1;191:109-17.
39. Marshall E, Claes L, Bouman WP, et al. Non-suicidal self-injury and suicidality in trans people: a systematic review of the literature. International review of psychiatry. 2016 Jan 2;28(1):58-69.
40. Marshal MP, Dietz LJ, Friedman MS, et al. Suicidality and depression disparities between sexual minority and heterosexual youth: A meta-analytic review. Journal of adolescent health. 2011 Aug 1;49(2):115-23.
41. E Mann G, Taylor A, Wren B, et al. Review of the literature on self-injurious thoughts and behaviours in gender-diverse children and young people in the United Kingdom. Clinical child psychology and psychiatry. 2018:1359104518812724.
42. Shamseer L, Moher D, Clarke M, et al. Preferred reporting items for systematic review and meta-analysis protocols (PRISMA-P) 2015: elaboration and explanation. Bmj. 2015 Jan 2;349:g7647.
43. Wells G, Shea B, O'Connell D, et al. Newcastle-Ottawa quality assessment scale cohort studies. 2011. http://www.ohri.ca/programs/clinical_epidemiology/oxford. asp.
44. Herzog R, Álvarez-Pasquin MJ, Díaz C, et al. Are healthcare workers' intentions to vaccinate related to their knowledge, beliefs and attitudes? A systematic review. BMC public health. 2013 Dec;13(1):154.
45. Beck AT, Steer RA, Ranieri WF. Scale for suicide ideation: psychometric properties of a self-report version. Journal of clinical psychology. 1988 Jul;44(4):499-505.
46. Bostrom AG, Cronbach J. Development of a clinical instrument to estimate suicide risk. Am J Psychiatry. 1985 Jun 6;142(6):680-6.
47. Muehlenkamp JJ, Gutierrez PM, Osman A, et al. Validation of the Positive and Negative Suicide Ideation (PANSI) Inventory in a diverse sample of young adults. Journal of Clinical Psychology. 2005 Apr;61(4):431-45.
48. Posner K, Brown GK, Stanley B, et al. The Columbia–Suicide Severity Rating Scale: initial validity and internal consistency findings from three multisite studies with adolescents and adults. American Journal of Psychiatry. 2011 Dec;168(12):1266-77.
49. Sansone RA, Wiederman MW, Sansone LA. The self-harm inventory (SHI): Development of a scale for identifying self-destructive behaviors and borderline personality disorder. Journal of clinical psychology. 1998 Nov;54(7):973-83.

For peer review only - http://bmjopen.bmj.com/site/about/guidelines.xhtml

50. Nock MK, Holmberg EB, Photos VI, Michel BD. Self-Injurious Thoughts and Behaviors Interview: Development, reliability, and validity in an adolescent sample.
51. Boudewyn AC, Liem JH. Mental health history form. 1995. *Unpublished measure, University of Massachusetts Boston*.
52. Taylor PJ, Dhingra K, Dickson JM, et al. Psychological correlates of self-harm within gay, lesbian and bisexual UK University students. Archives of suicide research. 2018 Sep 28:1-6.
53. Knipe D, Williams AJ, Hannan-Swain S, et al. Psychiatric morbidity and suicidal behaviour in low and middle-income countries: A systematic review and meta-analysis. (*in press*)
54. Harbord RM, Egger M, Sterne JA. A modified test for small-study effects in meta-analyses of controlled trials with binary endpoints. Statistics in medicine. 2006 Oct 30;25(20):3443-57.
55. Arcelus J, Claes L, Witcomb GL, et al. Risk factors for non-suicidal self-injury among trans youth. The Journal of Sexual Medicine. 2016 Mar 1;13(3):402-12.
56. Deeks JJ, Higgins JP, Altman DG. Analysing data and undertaking meta-analyses. Cochrane handbook for systematic reviews of interventions: Cochrane book series. 2008 Sep 26:243-96.
57. Popay J, Roberts H, Sowden A, et al. Guidance on the conduct of narrative synthesis in systematic reviews. A product from the ESRC methods programme Version. 2006 Apr 1;1:b92.

For peer review only

PRISMA-P 2015 Checklist

This checklist has been adapted for use with protocol submissions to *Systematic Reviews* from Table 3 in Moher D et al: Preferred reporting items for systematic review and meta-analysis protocols (PRISMA-P) 2015 statement. *Systematic Reviews* 2015 4:1

Section/topic	#	Checklist item	Information reported		Line number(s)
			Yes	No	
**ADMINISTRATIVE INFORMATION**					
**Title**					
Identification	1a	Identify the report as a protocol of a systematic review	√	☐	1
Update	1b	If the protocol is for an update of a previous systematic review, identify as such	☐	√	
**Registration**	2	If registered, provide the name of the registry (e.g., PROSPERO) and registration number in the Abstract	√	☐	47
**Authors**					
Contact	3a	Provide name, institutional affiliation, and e-mail address of all protocol authors; provide physical mailing address of corresponding author	√	☐	3-19
Contributions	3b	Describe contributions of protocol authors and identify the guarantor of the review	√	☐	237-239
**Amendments**	4	If the protocol represents an amendment of a previously completed or published protocol, identify as such and list changes; otherwise, state plan for documenting important protocol amendments	☐	√	
**Support**					
Sources	5a	Indicate sources of financial or other support for the review	√	☐	242-243
Sponsor	5b	Provide name for the review funder and/or sponsor	√	☐	242
Role of sponsor/funder	5c	Describe roles of funder(s), sponsor(s), and/or institution(s), if any, in developing the protocol	√	☐	242-243
**INTRODUCTION**					
**Rationale**	6	Describe the rationale for the review in the context of what is already known	√	☐	56-108
**Objectives**	7	Provide an explicit statement of the question(s) the review will address with reference to participants, interventions, comparators, and outcomes (PICO)	√	☐	109-110

For peer review only

BioMed Central The Open Access Publisher

2

Section/topic	#	Checklist item	Information reported Yes	Information reported No	Line number(s)
**METHODS**					
**Eligibility criteria**	8	Specify the study characteristics (e.g., PICO, study design, setting, time frame) and report characteristics (e.g., years considered, language, publication status) to be used as criteria for eligibility for the review	√	☐	120-148
**Information sources**	9	Describe all intended information sources (e.g., electronic databases, contact with study authors, trial registers, or other grey literature sources) with planned dates of coverage	√	☐	151-153; 176-177
**Search strategy**	10	Present draft of search strategy to be used for at least one electronic database, including planned limits, such that it could be repeated	√	☐	Sup file 2
STUDY RECORDS					
Data management	11a	Describe the mechanism(s) that will be used to manage records and data throughout the review	√	☐	165-187
Selection process	11b	State the process that will be used for selecting studies (e.g., two independent reviewers) through each phase of the review (i.e., screening, eligibility, and inclusion in meta-analysis)	√	☐	160-164
Data collection process	11c	Describe planned method of extracting data from reports (e.g., piloting forms, done independently, in duplicate), any processes for obtaining and confirming data from investigators	√	☐	153-154; 165-187
**Data items**	12	List and define all variables for which data will be sought (e.g., PICO items, funding sources), any pre-planned data assumptions and simplifications	√	☐	181-187
**Outcomes and prioritization**	13	List and define all outcomes for which data will be sought, including prioritization of main and additional outcomes, with rationale	√	☐	136-146
**Risk of bias in individual studies**	14	Describe anticipated methods for assessing risk of bias of individual studies, including whether this will be done at the outcome or study level, or both; state how this information will be used in data synthesis	√	☐	188-190
DATA					
**Synthesis**	15a	Describe criteria under which study data will be quantitatively synthesized	√	☐	191-212
	15b	If data are appropriate for quantitative synthesis, describe planned summary measures, methods of handling data, and methods of combining data from studies, including any planned exploration of consistency (e.g., I^2, Kendall's tau)	√	☐	194-200
	15c	Describe any proposed additional analyses (e.g., sensitivity or subgroup analyses, meta-regression)	√	☐	201-206
	15d	If quantitative synthesis is not appropriate, describe the type of summary planned	√	☐	209-213
**Meta-bias(es)**	16	Specify any planned assessment of meta-bias(es) (e.g., publication bias across studies, selective	√	☐	199-200

For peer review only

BioMed Central
The Open Access Publisher

Section/topic	#	Checklist item	Information reported		Line number(s)
			Yes	No	
		reporting within studies)			
**Confidence in cumulative evidence**	17	Describe how the strength of the body of evidence will be assessed (e.g., GRADE)	√	☐	188-190

For peer review only

BioMed Central The Open Access Publisher

SUPPLEMENTARY FILE 2

Search strategy terms:

(self-harm OR self harm* OR self-injur* OR "self injur*" OR self-cut* OR self-destruct* OR "self destruct*" OR "nonsuicidal self-injur*" OR "non-suicidal self injur*" OR "deliberate self harm" OR "deliberate self-harm" OR DSH OR "self-mutil*" OR overdos* OR self-inflicted injur* OR "self inflicted injur*" OR suicid* OR "parasuicid*" OR para-suicid* OR parasuicid* OR suicidal behav* OR suicide* OR "life-threatening behavio*" OR "suicide ideat*" OR "suicide attempt*" OR "attempted suicide*" OR NSSI)

AND

(moderat* OR mediat* OR "risk facto*" OR mechan* OR predict* OR pathway OR interact* OR "protective facto*" OR facto* OR influence OR correlate* OR precurs* OR "causal facto*")

AND

(transgender* OR transsexual* OR "gender nonconforming" OR "gender identity disorder" OR "gender dysphoria" OR "gender minority" OR lesbian*OR gay* OR bisexual* OR "sexual minority" OR "same-sex" OR homosexual* OR "homosexuality, male" OR "homosexuality, female" OR "gender identity" OR non-heterosexual* OR "non heterosexual*"OR homosexuality OR queer* OR questioning OR "non-binary" OR "non binary" OR "LGBT*" OR "sexual dissident*" OR "sexual and gender minorities" OR "gender variant" OR gender-variant OR genderqueer OR intersex OR "minority groups" OR "TGNC" OR "transgender and gender nonconforming")

AND

(Child* OR adolesc* OR "young people" OR kid* OR pupils OR youth OR juvenile OR "young adult*" OR "young person" OR minor*)

For peer review only

Example of highly sensitive search strategy used in EMBASE

*Search terms (AND, OR, NOT) and truncation (wildcard characters like *)*	1. exp automutilation/<br>2. exp suicide/<br>3. exp suicide attempt/<br>4. exp suicidal ideation/<br>5. 1 or 2 or 3 or 4<br>6. exp risk factor/<br>7. exp "sexual and gender minority"/<br>8. exp homosexuality/<br>9. exp bisexuality/<br>10. exp transgender/<br>11. exp LGBT people/<br>12. exp gender identity/<br>13. 7 or 8 or 9 or 10 or 11 or 12<br>14. exp adolescent/<br>15. exp child/<br>16. young people.mp.<br>17. exp young adult/<br>18. exp juvenile/<br>19. 14 or 15 or 16 or 17 or 18<br>20. 5 and 6 and 13 and 19

For peer review only

A systematic review protocol examining risk factors for self-harm and suicide in LGBTQ+ young people

Journal:	*BMJ Open*
Manuscript ID	bmjopen-2019-031541.R1
Article Type:	Protocol
Date Submitted by the Author:	06-Aug-2019
Complete List of Authors:	Williams, A. Jess; University of Birmingham, School of Psychology<br>Arcelus, J; University of Nottingham , School of Medicine<br>Townsend, Ellen; University of Nottingham, School of Psychology<br>Michail, Maria; University of Birmingham, School of Psychology
**Primary Subject Heading**:	Mental health
Secondary Subject Heading:	Public health, Research methods
Keywords:	Suicide & self-harm < PSYCHIATRY, gender identity, young people, sexual orientation

SCHOLARONE™
Manuscripts

A systematic review protocol examining risk factors for self-harm and suicide in LGBTQ+ young people

A. Jess Williams*[1,2] (a.williams.10@pgr.bham.ac.uk), Jon Arcelus[3] (jon.arcelus@nottingham.ac.uk), Ellen Townsend[2] (ellen.townsend@nottingham.ac.uk), Maria Michail[1] (m.michail@bham.ac.uk)

[1]Institute for Mental Health, School of Psychology, University of Birmingham, UK

[2]Self-Harm Research Group, School of Psychology, University of Nottingham, UK

[3]Institute of Mental Health, School of Medicine, University of Nottingham, UK

* Corresponding author

52 Pritchatts Road,

School of Psychology

Institute for Mental Health

University of Birmingham

B15 2TT

a.williams.10@pgr.bham.ac.uk

Tel. +(44)121 4142855

Word count: 2396 (excluding title page, abstract, references, figures and tables)

Key words:

Self-harm, suicide, sexual orientation, gender identity, young people

For peer review only - http://bmjopen.bmj.com/site/about/guidelines.xhtml

ABSTRACT

Introduction: Young people who identify as LGBTQ+ are at increased risk for self-harm, suicide ideation and behaviours. However, there has yet to be a comprehensive understanding of what risk factors influence these behaviours within LGBTQ+ young people as a whole. The purpose of this systematic review is to examine risk factors associated with self-harm, suicidal ideation and behaviour in Lesbian, Gay, Bisexual, Transgender, Queer or Questioning (LGBTQ+) young people.

Methods and analysis: A systematic review will be conducted, conforming to the reporting guidelines of the Preferred Reporting Items for Systematic Reviews and Meta-Analyses (PRISMA) statement recommendations. Electronic databases (MEDLINE, Scopus, EMBASE, PsycINFO, and Web of Science) will be systematically searched for cross-sectional, prospective, longitudinal, cohort, and case-control designs which examine risk factors for self-harm and/or suicidal ideation and behaviour in LGBTQ+ young people (aged 12-25 years). Only studies published in English will be included. No date restrictions will be applied. Study quality assessment will be conducted using the original and modified Newcastle-Ottawa Scales. Meta-analysis or narrative synthesis will be utilised, dependent on findings.

Ethics and dissemination: This is a systematic review of published literature and thereby ethical approval was not sought. The review will be submitted to a peer-reviewed journal, be publicly disseminated at conferences focusing on mental health, self-harm and suicide prevention. The findings will also be shared through public engagement and involvement, particularly those related to young LGBTQ+ individuals.

PROSPERO registration number: CRD42019130037

STRENGTHS AND LIMITATIONS OF THIS STUDY

- This is the first systematic review of risk factors for both self-harm as an individual behaviour, and suicidal ideation and suicidal behaviour among LGBTQ+ young people.
- The protocol has been written following the PRISMA-P guidelines (2015).
- The robust search strategy was conducted with input from an academic skills specialist to ensure replication across all databases, and capture a large range of papers.
- The systematic review will follow the Cochrane Review guidelines.
- A limitation of this systematic review is the exclusion of studies which are not published in English; this may bias findings against non-English speaking countries.

For peer review only - http://bmjopen.bmj.com/site/about/guidelines.xhtml

INTRODUCTION

Suicide, intentionally ending one's own life[1], is one of the leading causes of death within young people[2-3]. Globally, suicide accounts for around 131,441 deaths between the ages of 15-24[2]. Rates of self-harm, the self-injury or poisoning of one's self irrespective of suicidal intent[4], are on the rise among young people [5]. Data from the 2014 UK Adult Morbidity Survey states that 25.7% of women and 9.7% of men were likely to have self-harmed between the ages of 16-24 years[6]. Approximately 50% of those who die by suicide have previously self-harmed, making self-harm one of the strongest predictors of suicide[7-9].

Among people who identify under the umbrella term LGBTQ+ (Lesbian, Gay, Bisexual, Transgender, Queer or Questioning), this being how one self-identifies as their sex, sexuality, gender identity and gender expression[10], there are elevated rates of suicidal ideation (thoughts of self-harm, the consideration or planning of suicide[1]) and suicidal behaviour[11-13]. King et al's 2008 systematic review found that lesbian, gay and bisexual (LGB) individuals were found to be at higher risk for both suicidal behaviour and ideation compared to heterosexuals[11]. Internationally, this has been estimated to be between four to eight times higher in LGB and transgender (T) young people when compared to the heterosexual and cisgender peers[14]. While as a broad group, sexual and gender minorities have high levels of suicidal ideation and behaviour, among transgender people (individuals who do not present or identify with their sex assigned at birth[15]) these rates are even greater. Indeed, lifetime risk for suicide attempt is estimated to be between 22-43%, with 9-10% having made an attempt in the past 12 months[16-20].

Previous research has examined potential risk factors for self-harm and suicide in young people in general populations. These risk factors range from demographic characteristics such as being female, being a younger adult or adolescent, having fewer qualifications[21], to childhood abuse and neglect[22-23]. Additional risks such as bullying and academic pressure have been linked to suicidal behaviour in young people[24], as well as any mental health disorder[25]. Other factors which relate to the risk of self-harm repetition include troubled relationships with family members, social isolation, poor academic performance, alcohol and drug misuse, and depression[26].

Given the heightened risk of suicide attempt within LGBTQ+[11-13, 27], it would be pertinent to explore which factors specifically pose as a risk to this population. Although some of these risk factors may overlap with those from non-LGBTQ+ populations (e.g. depression, substance or alcohol misuse)[11, 28], there are also unique risk factors to sexual and gender minorities. For example, Clements-Nolle et al[28] stated that experiences of abuse, discrimination or harassment due to an individual's gender identity or presentation were also linked to high levels of attempted suicide. Meyer[29-30] suggested that additional risk within homosexual communities may be related to the high levels of stigma, prejudice and discrimination which thereby impacts individual's mental wellbeing. Victimisation or discrimination due to sexual orientation and gender identity is particularly common within LGBTQ+ populations[31-34]. In addition, among LGBTQ+ young people these experiences were found to be significantly associated with suicidal ideation[35-36]. It is suggested that young people may internalise these experiences of public stigma in relation to being LGBTQ+, known as internalised homophobia [37], which can lead to adverse impacts on their self-perception and beliefs, which could then enhance suicidal ideation, behaviour or self-harm[38].

For peer review only - http://bmjopen.bmj.com/site/about/guidelines.xhtml

Why this review is important

The goal of this systematic review is to comprehensively examine risk factors for self-harm and suicide ideation and behaviour within LGBTQ+ young people. Identifying and understanding how these factors relate to self-harm and suicide could allow future research to address specific risks and streamline potential studies to target the mental health needs of this population. Previous reviews regarding various dimensions of self-harm[22-23, 39-41] have focused on a specific subgroup, such as the prevalence within transgender population[42] or sexual orientation[43]. Whereas, few have considered both self-harm and suicide in relation to LGBTQ+ populations as a broad group[11, 43-45]. Thus, this review not only takes a dimensional approach to include self-harmful thoughts and behaviour with and without intent of suicide, thereby covering a wide spectrum of suicide and self-harm, but also includes all sexual and gender minorities.

Objectives:

1) To examine risk factors associated with self-harm and suicide ideation and behaviour in LGBTQ+ young people.
2) To examine whether there is a difference between sexual orientation minority young people and gender identity minority young people in the type of risk factors for self-harm, suicidal ideation and behaviour.

METHODS

A systematic review of empirical quantitative studies which examine risk factors for self-harm and suicidal ideation and behaviour in LGBTQ+ young people will be conducted. The search considered all studies up to the 1st of April 2019, a summary of eligibility criteria is shown in table 1. This protocol follows the Preferred Reporting Items for Systematic Reviews and Meta-Analyses Protocol (PRISMA-P) guidelines[46], presented in the supplementary file 1. Study quality will be assessed by the Newcastle-Ottawa Scale (NOS)[47] and the adapted version for cross-sectional studies[48]. The protocol for this article has been registered with PROSPERO: CRD42019130037.

Eligibility criteria: see table 1 for summary:

Types of studies: These include empirical quantitative peer reviewed studies following cross-sectional, prospective, longitudinal, cohort, and case-control designs which examine risk factors for self-harm and/or suicidal ideation and behaviour in LGBTQ+ young people. Risk factors being identified as significant predictors, mediators or moderators which influence a self-harm or suicidal outcome. These papers may specifically focus on one subgroup of the LGBTQ+ umbrella (e.g. transgender) or look across groups (e.g. LGB). Mixed-method papers which have applicable extractable information will also be included. Grey literature such as theses, dissertations or conference proceedings will not be included. Commentary, reviews, editorial or opinion pieces will also be excluded. All included studies must be available as full-text and peer-reviewed in the English language.

Types of participants: Participants between the ages of 12-25 years old, who identify as LGBTQ+ (Lesbian, Gay, Bisexual, Transgender, Queer or Questioning) referring to how one self-identifies as their sex, sexuality, gender identity and gender expression[10]. Participants will also have had

For peer review only - http://bmjopen.bmj.com/site/about/guidelines.xhtml

experiences of self-harm (self-injury or poisoning irrespective of suicidal intention[4]), suicide ideation (this can include thoughts of self-harm, the consideration or planning of a suicidal attempt e.g. method[1]) or suicidal behaviour (intentionally trying to end one's own life[1]). Alternatively, if participants met the above inclusion criteria as a subgroup, this data will be extracted.

Types of outcome measures: Studies will be included if they used a measure for self-harm, suicidal ideation or suicidal behaviour. A binary variable of self-harm, suicidal ideation and suicidal attempt will be taken as described by study authors, and any standardised rating scale for assessing the presence of self-harm and suicidal ideation and behaviour. This can be specific tools for considering suicide e.g. Beck Scale for Suicide Ideation (SSI)[49]; Motto's Risk Estimator for Suicide[50]; Positive and Negative Suicide Ideation (PANSI)[51]; Columbia-Suicide Severity Rating Scale[52]; or self-harm e.g. Self-Harm Inventory (SHI)[53]; Inventory of Statements about Self-injury (ISAS)[38]; Self-Injurious Thoughts and Behaviour Interview (SITBI) Short Form[54]. Also included will be self-harm or suicide items from general scales such as items 102-105 from the Mental Health History Form[55] or individual questions regarding self-harm or suicide had ever been considered or acted upon[56] and clinician reports.

Table 1: Inclusion and exclusion criteria for papers

**Inclusion Criteria**	**Exclusion criteria**
- Peer reviewed studies.<br>- Any geographical location.<br>- English language.<br>- Empirical quantitative studies, following cross-sectional, prospective, longitudinal, cohort and case-control designs.<br>- Studies must consider factors associated with or predictive of self-harm, suicidal ideation or suicidal behaviour.<br>- Participants must be young people (12-25 years).<br>- Participants that are identified or self-identified as any sexual or gender minority or member of LGBTQ+.<br>- Participants that have had an experience of self-harm, suicidal ideation or behaviour.	- Non-peer reviewed literature.<br>- Not English language.<br>- Grey literature such as theses, dissertation or conference proceedings.<br>- Articles such as commentaries, reviews, editorial or opinion pieces.<br>- Empirical qualitative studies.<br>- Studies not considering risk factors.<br>- Sample not aged between 12 and 25 years, e.g. adults 26 years and above or children 12 years and under.<br>- Participants who are identified as heterosexual or not part of sexual or gender minority.<br>- Participants who have no experience of self-harm, suicidal ideation or suicidal behaviour.

Search strategy:

The search was limited to English language and was ran up to the 1st April 2019. No date restrictions were applied. The following electronic bibliographic databases were searched: MEDLINE, Scopus, EMBASE, PsycINFO, and Web of Science for peer-reviewed publications which examine risk factors for self-harm or suicide within LGBTQ+ young people. The search strategy can be found in the supplementary file 2. This was developed in collaboration with an academic skill specialist from the University of Birmingham library to ensure the robustness of the search. Reference lists of eligible

For peer review only

For peer review only - http://bmjopen.bmj.com/site/about/guidelines.xhtml

papers and conduct citation searches of key papers were explored to identify additional reports. Before the final draft of the systematic review is completed, a second search will be conducted to allow for any additional studies to be identified.

Study records:

Selection process: The search strategy will retrieve titles and/or abstracts which will be screened by two independent researchers to identify studies which potentially meet the inclusion criteria outlined above. The full-texts of the studies will be retrieved and independently assessed for eligibility. Any disagreement or uncertainty over the eligibility of particular studies will be resolved through discussion by two independent researchers.

Data management and collection: Rayyan QCRI (https://rayyan.qcri.org/welcome), the online systematic review tool, will be used to manage and screen all retrieved papers. AJW will be the solo team member responsible for adding or amending paper records in Rayyan, as well as identifying and removing duplicates. A second independent reviewer will be given access to all titles and abstracts, within Rayyan. They will be able to make their own decision as to whether to include or exclude a paper, blind to AJW's decisions. AJW will also be blind to the decisions of the second reviewer until all titles and abstracts are reviewed. Rayyan will provide information on the original number of titles screened, duplications, those excluded at this stage, and those included titles at this stage.

Those papers which reach full-text screening will be managed within Zotero (https://www.zotero.org/), the bibliographic software. These will be sectioned by those included at full-text screening, and final inclusion. Reason for exclusion will be reported for each paper. AJW will be responsible for liaising with inter-library loans and obtaining the full-text papers. Full-text screening will again be conducted by two researchers, blind to each other's decisions, who will resolve disagreements through discussion. If an agreement cannot be reached regarding a paper, this will be rated by a third researcher.

A pre-piloted standardised data extraction tool will be adapted and used on included studies[57]. Extracted information will include: 1) author and publication date; 2) study design and setting; 3) characteristics of participants (age, gender identity), and the studies' inclusion and exclusion criteria; 4) method of harm (self-harm, suicidal behaviour or ideation); 5) factors associated with or predictive of self-harm or suicidal ideation and behaviour (including clinician diagnosis, subscales or validated scales used to assess these items); 6) information relating to risk of bias for the individual study.

Risk of bias (quality assessment): The overall quality of each study will be evaluated by two independent researchers using the Newcastle-Ottawa Scale (NOS)[47] and the Newcastle-Ottawa Scale adapted for cross-sectional studies[48]. Alternative study designs which are not covered in these versions of NOS, will be assessed using a pre-piloted version developed for a previous systematic review[57]. The level of agreement will be demonstrated by Cohen's Kappa and a Prevalence and Bias-Adjusted Kappa (PABAK) score.

For peer review only - http://bmjopen.bmj.com/site/about/guidelines.xhtml

Data synthesis and assessment of heterogeneity: Descriptive results of each included study will be presented in a table format, such as details regarding study design, participants, self-harm or suicidal ideation and behaviour methods and measures, identified factors and tools used to assess these.

A meta-analysis of risk factors for self-harm and suicidal ideation and behaviour using RevMan 5 (https://community.cochrane.org/help/tools-and-software/revman-5/revman-5-download) may be conducted by pooling data on risk factors using a random-effects model with the assumption that populations have various effect sizes and weights studies from in- and between-study variance. This will consider the extent of heterogeneity, which will be determined using the I^2 statistic. From this, a summary effect will be presented, associated 95% CI and *p* values. Odds ratio (OR) with 95% confidence intervals will present the overall synthesised measure of effect size. Publication bias will be assessed using funnel plots and Harbord-Egger test[58].

Additionally, consideration will be given to subgroup analysis if data allows, exploring heterogeneity by sexual and gender minorities; such that these studies are split by LGBQ and transgender; as even within this population it is recognised that the transgender population have high levels of suicide risk and self-harm[42, 59]. This will allow us to determine which risk factors are similar within sexual and gender minorities, and those that unique to the transgender population. Further examination of risk factors will be conducted by age difference, split by 12-18years and 19-25years.

The quality of the included studies will be considered when synthesising and analysing the findings in terms of the strength of evidence which they provide. A sensitivity analysis will be run to assess the robustness of the studies [60]. If statistical pooling is not possible, findings will follow a narrative synthesis as suggested by ERSC guidelines [61]. The analysis will take place over four steps; 1) developing a theory of which factors influence LGBTQ+ young people, 2) synthesising the findings, 3) exploring whether there are relationships within the data and what these might be, 4) assessing the robustness of the synthesis.

Patient and Public Involvement: No patients involved.

ETHICS AND DISSEMINATION

This is a systematic review of published literature and thereby ethical approval was not sought. It will be submitted to a peer-reviewed journal, be publicly disseminated at conferences focusing on mental health, self-harm and suicide prevention. Findings will also be shared through public engagement and involvement, particularly considering young LGBTQ+ individuals.

DISCUSSION

This systematic review will be the first to provide a rich, holistic account of the existing evidence of risk factors for self-harm and suicidality within a broad sample of LGBTQ+ young people. The synthesis of these findings will assess the prevalence of particular factors which impact this population, which may not be relevant to non-sexual or gender minority young people. Acknowledging the similarities and differences of risk factors between sexual orientation and gender

For peer review only - http://bmjopen.bmj.com/site/about/guidelines.xhtml

identity groups could be used as a valuable stepping stone when considering LGBTQ+ research and promoting more applicable research aims.

The quality and strength of the evidence will be rigorously assessed, which could be used to inform future research targeting these particular risks. This could ultimately help to inform self-harm and suicidality prevention within LGBTQ+ young people. It is anticipated that the findings of this review will be of interest to the wider academic and clinical community, policymakers, young people who identify as a sexual or gender minority, those with experience of self-harm or suicide.

AUTHORS CONTRIBUTIONS

AJW, JA, ET, and MM conceptualised the study. AJW developed the search strategy and conducted the literature search. AJW and MM wrote the first draft of the manuscript. AJW, JA, ET, and MM authors reviewed, edited, and approved the final manuscript.

FUNDING STATEMENT

This work was supported by the Midlands Graduate School ESRC DTP Joint Studentship awarded to A. Jess Williams. The funding body were not involved in the development of this protocol.

COMPETING INTERESTS

None declared.

For peer review only

For peer review only - http://bmjopen.bmj.com/site/about/guidelines.xhtml

REFERENCES

1. American Psychiatric Association. Diagnostic and statistical manual of mental disorders (DSM-5®). American Psychiatric Pub; 2013 May 22.
2. World Health Organization. Suicide across the world. 2016. Available from: https://www.who.int/mental_health/prevention/suicide/suicideprevent/en/. Accessed 7 January 2019.
3. Office for National Statistics. Suicides in the United Kingdom, 2017 Registration – ONS. 2018. Available at: https://www.ons.gov.uk/releases/suicidesintheuk2017registrations. Accessed 27 Feb. 2019.
4. National Collaborating Centre for Mental Health. Self-harm: Longer Term Management (NICE Clinical Guideline 133). London: National Institute for Clinical Excellence 2011.
5. Hawton, K., Saunders, K.E. and O'Connor, R.C., 2012. Self-harm and suicide in adolescents. *The Lancet*, *379*(9834), pp.2373-2382.
6. McManus S, Bebbington P, Jenkins R et al. Mental health and wellbeing in England: Adult Psychiatric Morbidity Survey 2014. Leeds: NHS Digital. 2016. Available at http://digital.nhs.uk/catalogue/PUB21748. [Accessed 27 Feb. 2019]
7. Sakinofsky I. Repetition of suicidal behaviour. In: Hawton K, and Van Heeringen K eds. The International Handbook of Suicide and Attempted Suicide. Chichester: John Wiley & Sons Ltd, 2000:385–404.
8. Hawton K, Bergen H, Cooper J, et al. Suicide following self-harm: findings from the Multicentre Study of self-harm in England, 2000–2012. Journal of Affective Disorders. 2015 April;(175):147-151.
9. Rodway C, Tham SG, Ibrahim S, et al. Suicide in children and young people in England: a consecutive case series. The Lancet Psychiatry. 2016 Aug 1;3(8):751-9.
10. Jourian TJ. Evolving nature of sexual orientation and gender identity. New Directions for Student Services. 2015 Dec;(152):11-23.
11. King M, Semlyen J, Tai SS, Killaspy H, Osborn D, Popelyuk D, Nazareth I. A systematic review of mental disorder, suicide, and deliberate self harm in lesbian, gay and bisexual people. BMC psychiatry. 2008 Dec;8(1):70.
12. Plöderl M, Wagenmakers EJ, Tremblay P, Ramsay R, Kralovec K, Fartacek C, Fartacek R. Suicide risk and sexual orientation: a critical review. Archives of Sexual Behavior. 2013 Jul 1;42(5):715-27.
13. Hottes TS, Bogaert L, Rhodes AE, et al. Lifetime prevalence of suicide attempts among sexual minority adults by study sampling strategies: a systematic review and meta-analysis. American journal of public health. 2016 May;106(5):e1-2.
14. Haas AP, Eliason M, Mays VM, Mathy RM, Cochran SD, D'Augelli AR, Silverman MM, Fisher PW, Hughes T, Rosario M, Russell ST. Suicide and suicide risk in lesbian, gay, bisexual, and transgender populations: Review and recommendations. Journal of homosexuality. 2010 Dec 30;58(1):10-51.
15. Richards C, Barker M. Sexuality and gender for mental health professionals: A practical guide. Sage; 2013 Oct 15.
16. Whittle S, Turner L, Combs R, et al. Transgender EuroStudy: Legal Survey and Focus on the Transgender Experience of Health Care. Brussels, Belgium: ILGA Europe; 2008. http://tgeu.org/sites/default/files/eurostudy.pdf. Accessed 26 March 2019

For peer review only - http://bmjopen.bmj.com/site/about/guidelines.xhtml

17. Bauer GR, Pyne J, Francino MC, et al. Suicidality among trans people in Ontario: implications for social work and social justice/La suicidabilité parmi les personnes trans en Ontario: implications en travail social et en justice sociale. Serv Soc. 2013;59:35–62.
18. Motmans J, de Biolley I, Debunne S. Being Transgender in Belgium: Mapping the Social and Legal Situation of Transgender People. Brussels: Institute for the Equality of Women and Men; 2010. http://igvmiefh. belgium.be/nl/binaries/34%20-%20Transgender_ENG_tcm336-99783.pdf. Accessed 26 March 2019
19. Grant JM, Mottet LA, Tanis J, et al. Injustice at Every Turn: A Report of the National Transgender Discrimination Survey. Washington, D.C.: National Center for Transgender Equality and National Gay and Lesbian Task Force; 2011. http://www.thetaskforce.org/downloads/reports/reports/ntds_full.pdf. Accessed 26 March 2019.
20. McNeil J, Bailey L, Ellis S, et al. Mental Health Study 2012. Edinburgh: Equality Network; 2012. http://www.scottishtrans.org/ Uploads/Resources/trans_mh_study.pdf. Accessed 26 March 2019.
21. Nock MK, Borges G, Bromet EJ, et al. Cross-national prevalence and risk factors for suicidal ideation, plans and attempts. The British Journal of Psychiatry. 2008 Feb;192(2):98-105.
22. Gratz KL. Risk factors for and functions of deliberate self-harm: An empirical and conceptual review. Clinical Psychology: Science and Practice. 2003 Jun;10(2):192-205.
23. Fliege H, Lee JR, Grimm A, et al. Risk factors and correlates of deliberate self-harm behavior: A systematic review. Journal of psychosomatic research. 2009 Jun 1;66(6):477-93.
24. Rodway C, Tham SG, Ibrahim S, et al. Suicide in children and young people in England: a consecutive case series. The Lancet Psychiatry. 2016 Aug 1;3(8):751-9.
25. Gili, M., Castellvi, P., Vives, M., de la Torre-Luque, A., Almenara, J., Blasco, M.J., Cebrià, A.I., Gabilondo, A., Pérez-Ara, M.A., Lagares, C. and Parés-Badell, O., 2018. Mental disorders as risk factors for suicidal behavior in young people: A meta-analysis and systematic review of longitudinal studies. Journal of affective disorders.
26. Hawton K, James A. Suicide and deliberate self harm in young people. BMJ. 2005;330(7496):891–4.
27. Semlyen J, King M, Varney J, et al. Sexual orientation and symptoms of common mental disorder or low wellbeing: combined meta-analysis of 12 UK population health surveys. BMC psychiatry. 2016 Dec;16(1):67.
28. Clements-Nolle K, Marx R, Katz M. Attempted suicide among transgender persons: The influence of gender-based discrimination and victimization. Journal of homosexuality. 2006 Oct 11;51(3):53-69.
29. Meyer IH. Minority stress and mental health in gay men. Journal of health and social behavior.1995 Mar;1:38-56.
30. Meyer IH. Prejudice, social stress, and mental health in lesbian, gay, and bisexual populations: conceptual issues and research evidence. Psychological bulletin. 2003 Sep;129(5):674.
31. Cochran SD, Mays VM. Lifetime prevalence of suicide symptoms and affective disorders among men reporting same-sex sexual partners: results from NHANES III. American journal of public health. 2000 Apr;90(4):573.
32. Bontempo DE, d'Augelli AR. Effects of at-school victimization and sexual orientation on lesbian, gay, or bisexual youths' health risk behavior. Journal of Adolescent Health. 2002 May 1;30(5):364-74.

For peer review only - http://bmjopen.bmj.com/site/about/guidelines.xhtml

33. Grossman AH, D'augelli AR, Frank JA. Aspects of psychological resilience among transgender youth. Journal of LGBT Youth. 2011 Mar 29;8(2):103-15.
34. Brandelli Costa A, Pasley A, Machado WD, et al. The experience of sexual stigma and the increased risk of attempted suicide in young Brazilian people from low socioeconomic group. Frontiers in psychology. 2017 Feb 22;8:192.
35. Kelleher C. Minority stress and health: Implications for lesbian, gay, bisexual, transgender, and questioning (LGBTQ) young people. Counselling psychology quarterly. 2009 Dec 1;22(4):373-9.
36. Taliaferro LA, Muehlenkamp JJ. Nonsuicidal self-injury and suicidality among sexual minority youth: risk factors and protective connectedness factors. Academic pediatrics. 2017 Sep 1;17(7):715-22.
37. Puckett, J.A., Newcomb, M.E., Ryan, D.T., Swann, G., Garofalo, R. and Mustanski, B., 2017. Internalized homophobia and perceived stigma: A validation study of stigma measures in a sample of young men who have sex with men. *Sexuality Research and Social Policy*, *14*(1), pp.1-16.
38. McDermott E, Hughes E, Rawlings V. The social determinants of lesbian, gay, bisexual and transgender youth suicidality in England: a mixed methods study. Journal of Public Health. 2017 Oct 17;40(3):e244-51.
39. Klonsky ED. The functions of deliberate self-injury: A review of the evidence. Clinical psychology review. 2007 Mar 1;27(2):226-39.
40. Skegg K. Self-harm. The Lancet. 2005 Oct 22;366(9495):1471-83.
41. Edmondson AJ, Brennan CA, House AO. Non-suicidal reasons for self-harm: a systematic review of self-reported accounts. Journal of Affective Disorders. 2016 Feb 1;191:109-17.
42. Marshall E, Claes L, Bouman WP, et al. Non-suicidal self-injury and suicidality in trans people: a systematic review of the literature. International review of psychiatry. 2016 Jan 2;28(1):58-69.
43. Miranda-Mendizábal, A., Castellví, P., Parés-Badell, O., Almenara, J., Alonso, I., Blasco, M.J., Cebrià, A., Gabilondo, A., Gili, M., Lagares, C. and Piqueras, J.A., 2017. Sexual orientation and suicidal behaviour in adolescents and young adults: systematic review and meta-analysis. *The British Journal of Psychiatry*, *211*(2), pp.77-87.
44. Marshal MP, Dietz LJ, Friedman MS, et al. Suicidality and depression disparities between sexual minority and heterosexual youth: A meta-analytic review. Journal of adolescent health. 2011 Aug 1;49(2):115-23.
45. E Mann G, Taylor A, Wren B, et al. Review of the literature on self-injurious thoughts and behaviours in gender-diverse children and young people in the United Kingdom. Clinical child psychology and psychiatry. 2018:1359104518812724.
46. Shamseer L, Moher D, Clarke M, et al. Preferred reporting items for systematic review and meta-analysis protocols (PRISMA-P) 2015: elaboration and explanation. Bmj. 2015 Jan 2;349:g7647.
47. Wells G, Shea B, O'Connell D, et al. Newcastle-Ottawa quality assessment scale cohort studies. 2011. http://www.ohri.ca/programs/clinical_epidemiology/oxford. asp.
48. Herzog R, Álvarez-Pasquin MJ, Díaz C, et al. Are healthcare workers' intentions to vaccinate related to their knowledge, beliefs and attitudes? A systematic review. BMC public health. 2013 Dec;13(1):154.

For peer review only - http://bmjopen.bmj.com/site/about/guidelines.xhtml

49. Beck AT, Steer RA, Ranieri WF. Scale for suicide ideation: psychometric properties of a self-report version. Journal of clinical psychology. 1988 Jul;44(4):499-505.
50. Bostrom AG, Cronbach J. Development of a clinical instrument to estimate suicide risk. Am J Psychiatry. 1985 Jun 6;142(6):680-6.
51. Muehlenkamp JJ, Gutierrez PM, Osman A, et al. Validation of the Positive and Negative Suicide Ideation (PANSI) Inventory in a diverse sample of young adults. Journal of Clinical Psychology. 2005 Apr;61(4):431-45.
52. Posner K, Brown GK, Stanley B, et al. The Columbia–Suicide Severity Rating Scale: initial validity and internal consistency findings from three multisite studies with adolescents and adults. American Journal of Psychiatry. 2011 Dec;168(12):1266-77.
53. Sansone RA, Wiederman MW, Sansone LA. The self-harm inventory (SHI): Development of a scale for identifying self-destructive behaviors and borderline personality disorder. Journal of clinical psychology. 1998 Nov;54(7):973-83.
54. Nock MK, Holmberg EB, Photos VI, Michel BD. Self-Injurious Thoughts and Behaviors Interview: Development, reliability, and validity in an adolescent sample.
55. Boudewyn AC, Liem JH. Mental health history form. 1995. *Unpublished measure, University of Massachusetts Boston.*
56. Taylor PJ, Dhingra K, Dickson JM, et al. Psychological correlates of self-harm within gay, lesbian and bisexual UK University students. Archives of suicide research. 2018 Sep 28:1-6.
57. Knipe D, Williams AJ, Hannan-Swain S, et al. Psychiatric morbidity and suicidal behaviour in low and middle-income countries: A systematic review and meta-analysis. (*in press*)
58. Harbord RM, Egger M, Sterne JA. A modified test for small-study effects in meta-analyses of controlled trials with binary endpoints. Statistics in medicine. 2006 Oct 30;25(20):3443-57.
59. Arcelus J, Claes L, Witcomb GL, et al. Risk factors for non-suicidal self-injury among trans youth. The Journal of Sexual Medicine. 2016 Mar 1;13(3):402-12.
60. Deeks JJ, Higgins JP, Altman DG. Analysing data and undertaking meta-analyses. Cochrane handbook for systematic reviews of interventions: Cochrane book series. 2008 Sep 26:243-96.
61. Popay J, Roberts H, Sowden A, et al. Guidance on the conduct of narrative synthesis in systematic reviews. A product from the ESRC methods programme Version. 2006 Apr 1;1:b92.

For peer review only - http://bmjopen.bmj.com/site/about/guidelines.xhtml

PRISMA-P 2015 Checklist

This checklist has been adapted for use with protocol submissions to *Systematic Reviews* from Table 3 in Moher D et al: Preferred reporting items for systematic review and meta-analysis protocols (PRISMA-P) 2015 statement. *Systematic Reviews* 2015 **4**:1

Section/topic	#	Checklist item	Information reported: Yes	Information reported: No	Line number(s)
**ADMINISTRATIVE INFORMATION**					
**Title**					
Identification	1a	Identify the report as a protocol of a systematic review	√	☐	1
Update	1b	If the protocol is for an update of a previous systematic review, identify as such	☐	√	
**Registration**	2	If registered, provide the name of the registry (e.g., PROSPERO) and registration number in the Abstract	√	☐	48
**Authors**					
Contact	3a	Provide name, institutional affiliation, and e-mail address of all protocol authors; provide physical mailing address of corresponding author	√	☐	4-20
Contributions	3b	Describe contributions of protocol authors and identify the guarantor of the review	√	☐	250-253
**Amendments**	4	If the protocol represents an amendment of a previously completed or published protocol, identify as such and list changes; otherwise, state plan for documenting important protocol amendments	☐	√	
**Support**					
Sources	5a	Indicate sources of financial or other support for the review	√	☐	255-257
Sponsor	5b	Provide name for the review funder and/or sponsor	√	☐	256
Role of sponsor/funder	5c	Describe roles of funder(s), sponsor(s), and/or institution(s), if any, in developing the protocol	√	☐	257
**INTRODUCTION**					
**Rationale**	6	Describe the rationale for the review in the context of what is already known	√	☐	60-112
**Objectives**	7	Provide an explicit statement of the question(s) the review will address with reference to participants, interventions, comparators, and outcomes (PICO)	√	☐	113-118

BioMed Central
The Open Access Publisher

For peer review only - http://bmjopen.bmj.com/site/about/guidelines.xhtml

Section/topic	#	Checklist item	Information reported Yes	No	Line number(s)
**METHODS**					
**Eligibility criteria**	8	Specify the study characteristics (e.g., PICO, study design, setting, time frame) and report characteristics (e.g., years considered, language, publication status) to be used as criteria for eligibility for the review	√	☐	128-157
**Information sources**	9	Describe all intended information sources (e.g., electronic databases, contact with study authors, trial registers, or other grey literature sources) with planned dates of coverage	√	☐	160-168
**Search strategy**	10	Present draft of search strategy to be used for at least one electronic database, including planned limits, such that it could be repeated	√	☐	Sup file 2
STUDY RECORDS					
Data management	11a	Describe the mechanism(s) that will be used to manage records and data throughout the review	√	☐	175-197
Selection process	11b	State the process that will be used for selecting studies (e.g., two independent reviewers) through each phase of the review (i.e., screening, eligibility, and inclusion in meta-analysis)	√	☐	170-174
Data collection process	11c	Describe planned method of extracting data from reports (e.g., piloting forms, done independently, in duplicate), any processes for obtaining and confirming data from investigators	√	☐	175-197
**Data items**	12	List and define all variables for which data will be sought (e.g., PICO items, funding sources), any pre-planned data assumptions and simplifications	√	☐	191-197
**Outcomes and prioritization**	13	List and define all outcomes for which data will be sought, including prioritization of main and additional outcomes, with rationale	√	☐	146-156
**Risk of bias in individual studies**	14	Describe anticipated methods for assessing risk of bias of individual studies, including whether this will be done at the outcome or study level, or both; state how this information will be used in data synthesis	√	☐	198-203
DATA					
**Synthesis**	15a	Describe criteria under which study data will be quantitatively synthesized	√	☐	204-222
	15b	If data are appropriate for quantitative synthesis, describe planned summary measures, methods of handling data, and methods of combining data from studies, including any planned exploration of consistency (e.g., I^2, Kendall's tau)	√	☐	207-214
	15c	Describe any proposed additional analyses (e.g., sensitivity or subgroup analyses, meta-regression)	√	☐	215-220
	15d	If quantitative synthesis is not appropriate, describe the type of summary planned	√	☐	223-227
**Meta-bias(es)**	16	Specify any planned assessment of meta-bias(es) (e.g., publication bias across studies, selective	√	☐	221-223

BioMed Central The Open Access Publisher

Section/topic	#	Checklist item	Information reported		Line number(s)
			Yes	No	
		reporting within studies)			
**Confidence in cumulative evidence**	17	Describe how the strength of the body of evidence will be assessed (e.g., GRADE)	√	☐	198-203

For peer review only

BioMed Central The Open Access Publisher

SUPPLEMENTARY FILE 2

Search strategy terms:

(self-harm OR self harm* OR self-injur* OR "self injur*" OR self-cut* OR self-destruct* OR "self destruct*" OR "nonsuicidal self-injur*" OR "non-suicidal self injur*" OR "deliberate self harm" OR "deliberate self-harm" OR DSH OR "self-mutil*" OR overdos* OR self-inflicted injur* OR "self inflicted injur*" OR suicid* OR "parasuicid*" OR para-suicid* OR parasuicid* OR suicidal behav* OR suicide* OR "life-threatening behavio*" OR "suicide ideat*" OR "suicide attempt*" OR "attempted suicide*" OR NSSI)

AND

(moderat* OR mediat* OR "risk facto*" OR mechan* OR predict* OR pathway OR interact* OR "protective facto*" OR facto* OR influence OR correlate* OR precurs* OR "causal facto*")

AND

(transgender* OR transsexual* OR "gender nonconforming" OR "gender identity disorder" OR "gender dysphoria" OR "gender minority" OR lesbian*OR gay* OR bisexual* OR "sexual minority" OR "same-sex" OR homosexual* OR "homosexuality, male" OR "homosexuality, female" OR "gender identity" OR non-heterosexual* OR "non heterosexual*"OR homosexuality OR queer* OR questioning OR "non-binary" OR "non binary" OR "LGBT*" OR "sexual dissident*" OR "sexual and gender minorities" OR "gender variant" OR gender-variant OR genderqueer OR intersex OR "minority groups" OR "TGNC" OR "transgender and gender nonconforming")

AND

(Child* OR adolesc* OR "young people" OR kid* OR pupils OR youth OR juvenile OR "young adult*" OR "young person" OR minor*)

For peer review only

A systematic review protocol examining risk factors for self-harm and suicide in LGBTQ+ young people

Journal:	*BMJ Open*
Manuscript ID	bmjopen-2019-031541.R2
Article Type:	Protocol
Date Submitted by the Author:	16-Sep-2019

Complete List of Authors:	Williams, A. Jess; University of Birmingham, School of Psychology<br>Arcelus, J; University of Nottingham , School of Medicine<br>Townsend, Ellen; University of Nottingham, School of Psychology<br>Michail, Maria; University of Birmingham, School of Psychology

**Primary Subject Heading**:	Mental health
Secondary Subject Heading:	Public health, Research methods
Keywords:	Suicide & self-harm < PSYCHIATRY, gender identity, young people, sexual orientation, systematic review, protocol

A systematic review protocol examining risk factors for self-harm and suicide in LGBTQ+ young people

A. Jess Williams*[1,2] (a.williams.10@pgr.bham.ac.uk), Jon Arcelus[3] (jon.arcelus@nottingham.ac.uk), Ellen Townsend[2] (ellen.townsend@nottingham.ac.uk), Maria Michail[1] (m.michail@bham.ac.uk)

[1]Institute for Mental Health, School of Psychology, University of Birmingham, UK

[2]Self-Harm Research Group, School of Psychology, University of Nottingham, UK

[3]Institute of Mental Health, School of Medicine, University of Nottingham, UK

* Corresponding author

52 Pritchatts Road,

School of Psychology

Institute for Mental Health

University of Birmingham

B15 2TT

a.williams.10@pgr.bham.ac.uk

Tel. +(44)121 4142855

Word count: 2594 (excluding title page, abstract, references, figures and tables)

Key words:

Self-harm, suicide, sexual orientation, gender identity, young people

For peer review only

ABSTRACT

Introduction: Young people who identify as LGBTQ+ are at increased risk for self-harm, suicide ideation and behaviours. However, there has yet to be a comprehensive understanding of what risk factors influence these behaviours within LGBTQ+ young people as a whole. The purpose of this systematic review is to examine risk factors associated with self-harm, suicidal ideation and behaviour in Lesbian, Gay, Bisexual, Transgender, Queer or Questioning (LGBTQ+) young people.

Methods and analysis: A systematic review will be conducted, conforming to the reporting guidelines of the Preferred Reporting Items for Systematic Reviews and Meta-Analyses (PRISMA) statement recommendations. Electronic databases (MEDLINE, Scopus, EMBASE, PsycINFO, and Web of Science) will be systematically searched for cross-sectional, prospective, longitudinal, cohort, and case-control designs which examine risk factors for self-harm and/or suicidal ideation and behaviour in LGBTQ+ young people (aged 12-25 years). Only studies published in English will be included. No date restrictions will be applied. Study quality assessment will be conducted using the original and modified Newcastle-Ottawa Scales. Meta-analysis or narrative synthesis will be utilised, dependent on findings.

Ethics and dissemination: This is a systematic review of published literature and thereby ethical approval was not sought. The review will be submitted to a peer-reviewed journal, be publicly disseminated at conferences focusing on mental health, self-harm and suicide prevention. The findings will also be shared through public engagement and involvement, particularly those related to young LGBTQ+ individuals.

PROSPERO registration number: CRD42019130037

STRENGTHS AND LIMITATIONS OF THIS STUDY

- This is the first systematic review of risk factors for both self-harm as an individual behaviour, and suicidal ideation and suicidal behaviour among LGBTQ+ young people.
- The protocol has been written following the PRISMA-P guidelines (2015).
- The robust search strategy was conducted with input from an academic skills specialist to ensure replication across all databases, and capture a large range of papers.
- The systematic review will follow the Cochrane Review guidelines.
- A limitation of this systematic review is the exclusion of studies which are not published in English; this may bias findings against non-English speaking countries.

For peer review only - http://bmjopen.bmj.com/site/about/guidelines.xhtml

INTRODUCTION

Suicide, intentionally ending one's own life[1], is one of the leading causes of death within young people[2-3]. Globally, suicide accounts for around 131,441 deaths between the ages of 15-24[2]. Rates of self-harm, the self-injury or poisoning of one's self irrespective of suicidal intent[4], are on the rise among young people[5]. Data from the 2014 UK Adult Morbidity Survey states that 25.7% of women and 9.7% of men were likely to have self-harmed between the ages of 16-24 years[6]. Approximately 50% of those who die by suicide have previously self-harmed, making self-harm one of the strongest predictors of suicide[7-9].

Among people who identify under the umbrella term LGBTQ+ (Lesbian, Gay, Bisexual, Transgender, Queer or Questioning), this being how one self-identifies as their sex, sexuality, gender identity and gender expression[10], there are elevated rates of suicidal ideation (thoughts of self-harm, the consideration or planning of suicide[1]) and suicidal behaviour[11-13]. King et al's 2008 systematic review found that lesbian, gay and bisexual (LGB) individuals were found to be at higher risk for both suicidal behaviour and ideation compared to heterosexuals[11]. Internationally, this has been estimated to be between four to eight times higher in LGB and transgender (T) young people when compared to the heterosexual and cisgender peers[14]. While as a broad group, sexual and gender minorities have high levels of suicidal ideation and behaviour, among transgender people (individuals who do not present or identify with their sex assigned at birth[15]) these rates are even greater. Indeed, lifetime risk for suicide attempt is estimated to be between 22-43%, with 9-10% having made an attempt in the past 12 months[16-20].

Previous research has examined potential risk factors for self-harm and suicide in young people in general populations. These risk factors range from demographic characteristics such as being female, being a younger adult or adolescent, having fewer qualifications[21], to childhood abuse and neglect[22-23]. Additional risks such as bullying and academic pressure have been linked to suicidal behaviour in young people[9], as well as any mental health disorder[24]. Other factors which relate to the risk of self-harm repetition include troubled relationships with family members, social isolation, poor academic performance, alcohol and drug misuse, and depression[25].

Given the heightened risk of suicide attempt within LGBTQ+[11-13, 26], it would be pertinent to explore which factors specifically pose as a risk to this population. Although some of these risk factors may overlap with those from non-LGBTQ+ populations (e.g. depression, substance or alcohol misuse)[11, 27], there are also unique risk factors to sexual and gender minorities. For example, Clements-Nolle et al[27] stated that experiences of abuse, discrimination or harassment due to an individual's gender identity or presentation were also linked to high levels of attempted suicide. Meyer[28-29] suggested that additional risk within homosexual communities may be related to the high levels of stigma, prejudice and discrimination which thereby impacts individual's mental wellbeing. Victimisation or discrimination due to sexual orientation and gender identity is particularly common within LGBTQ+ populations[30-33]. In addition, among LGBTQ+ young people these experiences were found to be significantly associated with suicidal ideation[34-35]. It is suggested that young people may internalise these experiences of public stigma in relation to being LGBTQ+, known as internalised homophobia[36], which can lead to adverse impacts on their self-perception and beliefs, which could then enhance suicidal ideation, behaviour or self-harm[37].

For peer review only - http://bmjopen.bmj.com/site/about/guidelines.xhtml

Why this review is important

The goal of this systematic review is to comprehensively examine risk factors for self-harm and suicide ideation and behaviour within LGBTQ+ young people. Identifying and understanding how these factors relate to self-harm and suicide could allow future research to address specific risks and streamline potential studies to target the mental health needs of this population. Previous reviews regarding various dimensions of self-harm[22-23, 38-40] have focused on a specific subgroup, such as the prevalence within transgender population[41] or sexual orientation[42]. Whereas, few have considered both self-harm and suicide in relation to LGBTQ+ populations as a broad group[11, 42-44]. Thus, this review not only takes a dimensional approach to include self-harmful thoughts and behaviour with and without intent of suicide, thereby covering a wide spectrum of suicide and self-harm, but also includes all sexual and gender minorities.

Objectives:

1) To examine risk factors associated with self-harm and suicide ideation and behaviour in LGBTQ+ young people.
2) To examine whether there is a difference between sexual orientation minority young people and gender identity minority young people in the type of risk factors for self-harm, suicidal ideation and behaviour.

METHODS

A systematic review of empirical quantitative studies which examine risk factors for self-harm and suicidal ideation and behaviour in LGBTQ+ young people will be conducted. The search considered all studies up to the 1st of April 2019, a summary of eligibility criteria is shown in table 1. This protocol follows the Preferred Reporting Items for Systematic Reviews and Meta-Analyses Protocol (PRISMA-P) guidelines[45], presented in the supplementary file 1. Study quality will be assessed by the Newcastle-Ottawa Scale (NOS)[46] and the adapted version for cross-sectional studies[47]. The protocol for this article has been registered with PROSPERO: CRD42019130037.

Eligibility criteria: see table 1 for summary:

Types of studies: These include empirical quantitative peer reviewed studies following cross-sectional, prospective, longitudinal, cohort, and case-control designs which examine risk factors for self-harm and/or suicidal ideation and behaviour in LGBTQ+ young people. Risk factors being identified as significant predictors, mediators or moderators which influence a self-harm or suicidal outcome. These papers may specifically focus on one subgroup of the LGBTQ+ umbrella (e.g. transgender) or look across groups (e.g. LGB). Mixed-method papers which have applicable extractable information will also be included. Grey literature such as theses, dissertations or conference proceedings will not be included. Commentary, reviews, editorial or opinion pieces will also be excluded. All included studies must be available as full-text and peer-reviewed in the English language.

Types of participants: Participants between the ages of 12-25 years old, who identify as LGBTQ+ (Lesbian, Gay, Bisexual, Transgender, Queer or Questioning) referring to how one self-identifies as their sex, sexuality, gender identity and gender expression[10]. This age range was selected to mirror

For peer review only

For peer review only - http://bmjopen.bmj.com/site/about/guidelines.xhtml

papers within the field that consider young people up to the age of 25 years[9,48], with the lower limit extended to 12 years to include the adolescent period. Participants will also have had experiences of self-harm (self-injury or poisoning irrespective of suicidal intention[4]), suicide ideation (this can include thoughts of self-harm, the consideration or planning of a suicidal attempt e.g. method[1]) or suicidal behaviour (intentionally trying to end one's own life[1]). Alternatively, if participants met the above inclusion criteria as a subgroup, this data will be extracted.

Types of outcome measures: Studies will be included if they used a measure for self-harm, suicidal ideation or suicidal behaviour. The measurements of self-harm (any, with intent, without intent) and suicidal behaviour; attempt or death, will be taken as binary variables; present or absence. Continuous scales for suicidal ideation will be converted to binary variables by considering the original scale threshold. This will act as a cut-off point, if the population mean for ideation is above this threshold, ideation will be considered present, whereas below the threshold, ideation will be considered absent. The psychometric measures of these outcomes will be extracted for descriptive purposes. This can be specific tools for considering suicide e.g. Beck Scale for Suicide Ideation (SSI)[49]; Motto's Risk Estimator for Suicide[50]; Positive and Negative Suicide Ideation (PANSI)[51]; Columbia-Suicide Severity Rating Scale[52]; or self-harm e.g. Self-Harm Inventory (SHI)[53]; Inventory of Statements about Self-injury (ISAS)[38]; Self-Injurious Thoughts and Behaviour Interview (SITBI) Short Form[54]. Also included will be self-harm or suicide items from general scales such as items 102-105 from the Mental Health History Form[Boudewyn & Liem, 1995; Mental Health History Form] or individual questions regarding self-harm or suicide had ever been considered or acted upon[55] and clinician reports.

Table 1: Inclusion and exclusion criteria for papers

Inclusion Criteria	Exclusion criteria
- Peer reviewed studies.<br>- Any geographical location.<br>- English language.<br>- Empirical quantitative studies, following cross-sectional, prospective, longitudinal, cohort and case-control designs.<br>- Studies must consider factors associated with or predictive of self-harm, suicidal ideation or suicidal behaviour.<br>- Participants must be young people (12-25 years).<br>- Participants that are identified or self-identified as any sexual or gender minority or member of LGBTQ+.<br>- Participants that have had an experience of self-harm, suicidal ideation or behaviour.	- Non-peer reviewed literature.<br>- Not English language.<br>- Grey literature such as theses, dissertation or conference proceedings.<br>- Articles such as commentaries, reviews, editorial or opinion pieces.<br>- Empirical qualitative studies.<br>- Sample not aged between 12 and 25 years, e.g. adults 26 years and above or children 12 years and under.<br>- Participants who are identified as heterosexual or not part of sexual or gender minority.<br>- Participants who have no experience of self-harm, suicidal ideation or suicidal behaviour.

Search strategy:

For peer review only - http://bmjopen.bmj.com/site/about/guidelines.xhtml

The search was limited to English language and was run up to the 1st April 2019. No date restrictions were applied. The following electronic bibliographic databases were searched: MEDLINE, Scopus, EMBASE, PsycINFO, and Web of Science for peer-reviewed publications which examine risk factors for self-harm or suicide within LGBTQ+ young people. The search strategy can be found in the supplementary file 2. This was developed in collaboration with an academic skill specialist from the University of Birmingham library to ensure the robustness of the search. Reference lists of eligible papers and conduct citation searches of key papers were explored to identify additional reports. Before the final draft of the systematic review is completed, a second search will be conducted to allow for any additional studies to be identified.

Study records:

Selection process: The search strategy will retrieve titles and/or abstracts which will be screened by two independent researchers to identify studies which potentially meet the inclusion criteria outlined above. The full-texts of the studies will be retrieved and independently assessed for eligibility. Any disagreement or uncertainty over the eligibility of particular studies will be resolved through discussion by two independent researchers.

Data management and collection: Rayyan QCRI (https://rayyan.qcri.org/welcome), the online systematic review tool, will be used to manage and screen all retrieved papers. AJW will be the solo team member responsible for adding or amending paper records in Rayyan, as well as identifying and removing duplicates. A second independent reviewer will be given access to all titles and abstracts, within Rayyan. They will be able to make their own decision as to whether to include or exclude a paper, blind to AJW's decisions. AJW will also be blind to the decisions of the second reviewer until all titles and abstracts are reviewed. Rayyan will provide information on the original number of titles screened, duplications, those excluded at this stage, and those included titles at this stage.

Those papers which reach full-text screening will be managed within Zotero (https://www.zotero.org/), the bibliographic software. These will be sectioned by those included at full-text screening, and final inclusion. Reason for exclusion will be reported for each paper. AJW will be responsible for liaising with inter-library loans and obtaining the full-text papers. Full-text screening will again be conducted by two researchers, blind to each other's decisions, who will resolve disagreements through discussion. If an agreement cannot be reached regarding a paper, this will be rated by a third researcher.

A pre-piloted standardised data extraction tool will be adapted and used on included studies[56]. Extracted information will include: 1) author and publication date; 2) study design and setting; 3) characteristics of participants (age, gender identity), and the studies' inclusion and exclusion criteria; 4) method of harm (self-harm, suicidal behaviour or ideation); 5) factors associated with or predictive of self-harm or suicidal ideation and behaviour (including clinician diagnosis, subscales or validated scales used to assess these items); 6) information relating to risk of bias for the individual study.

Risk of bias (quality assessment): The overall quality of each study will be evaluated by two independent researchers using the Newcastle-Ottawa Scale (NOS)[47] and the Newcastle-Ottawa Scale adapted for cross-sectional studies[48]. Alternative study designs which are not covered in

For peer review only - http://bmjopen.bmj.com/site/about/guidelines.xhtml

these versions of NOS, will be assessed using a pre-piloted version developed for a previous systematic review[57]. The level of agreement will be demonstrated by Cohen's Kappa and a Prevalence and Bias-Adjusted Kappa (PABAK) score.

Data synthesis and assessment of heterogeneity: Included studies will be presented by grouping risk types together (e.g. demographic, psychosocial, psychiatric or mental health etc.). The descriptive characteristics of each will be presented in tabular format, and the risks and outcomes of which will be discussed using narrative synthesis, following the ESRC guidelines[58]. The analysis will take place over four steps; 1) developing a theory of which factors influence LGBTQ+ young people, 2) synthesising the findings, 3) exploring whether there are relationships within the data and what these might be, 4) assessing the robustness of the synthesis.

If possible, a meta-analysis of risk factors for self-harm and suicidal ideation and behaviour using RevMan 5 (https://community.cochrane.org/help/tools-and-software/revman-5/revman-5-download) may be conducted by pooling data on risk factors using a random-effects model with the assumption that populations have various effect sizes and weights studies from in- and between-study variance. These risks will be pooled based on the analysis which has taken place in the original paper, if a risk appears in more than one type of analysis it will be included in both analyses. These will consider the extent of heterogeneity, which will be determined using the I^2 statistic. From this, a summary effect will be presented, associated 95% CI and *p* values. Odds ratio (OR) with 95% confidence intervals will present the overall synthesised measure of effect size. Publication bias will be assessed using funnel plots and Harbord-Egger test[57].

Additionally, consideration will be given to subgroup analysis if data allows, exploring heterogeneity by sexual and gender minorities; such that these studies are split by LGBQ and transgender; as even within this population it is recognised that the transgender population have high levels of suicide risk and self-harm[42, 58]. This will allow us to determine which risk factors are similar within sexual and gender minorities, and those that unique to the transgender population. Further examination of risk factors will be conducted by age difference, split by 12-18years and 19-25years.

The quality of the included studies will be considered when synthesising and analysing the findings in terms of the strength of evidence which they provide. A sensitivity analysis will be run to assess the robustness of included studies[59], following the removal of papers which score lower than 70% on quality assessment scales.

Patient and Public Involvement: No patients involved.

ETHICS AND DISSEMINATION

This is a systematic review of published literature and thereby ethical approval was not sought. It will be submitted to a peer-reviewed journal, be publicly disseminated at conferences focusing on mental health, self-harm and suicide prevention. Findings will also be shared through public engagement and involvement, particularly considering young LGBTQ+ individuals.

For peer review only - http://bmjopen.bmj.com/site/about/guidelines.xhtml

DISCUSSION

This systematic review will be the first to provide a rich, holistic account of the existing evidence of risk factors for self-harm and suicidality within a broad sample of LGBTQ+ young people. The synthesis of these findings will assess the prevalence of particular factors which impact this population, which may not be relevant to non-sexual or gender minority young people. However, when pooling studies within a meta-analysis, it is possible that the variation between papers may cause a challenge for this synthesis. This may be related to the broad approach in which we're approaching the systematic review, nonetheless we feel that this approach offers many values. Such as, exploring the similarities and differences of risk factors by subgroup analysis, via sexual orientation and gender identity or age, could be used as a valuable stepping stone when considering LGBTQ+ research and promoting more applicable research aims.

The quality and strength of the evidence will be rigorously assessed, which could be used to inform future research targeting these particular risks. This could ultimately help to inform self-harm and suicidality prevention within LGBTQ+ young people. It is anticipated that the findings of this review will be of interest to the wider academic and clinical community, policymakers, young people who identify as a sexual or gender minority, those with experience of self-harm or suicide.

AUTHORS CONTRIBUTIONS

AJW, JA, ET, and MM conceptualised the study. AJW developed the search strategy and conducted the literature search. AJW and MM wrote the first draft of the manuscript. AJW, JA, ET, and MM authors reviewed, edited, and approved the final manuscript.

FUNDING STATEMENT

This work was supported by the Midlands Graduate School ESRC DTP Joint Studentship awarded to A. Jess Williams. The funding body were not involved in the development of this protocol.

COMPETING INTERESTS

None declared.

For peer review only - http://bmjopen.bmj.com/site/about/guidelines.xhtml

REFERENCES

1. American Psychiatric Association. Diagnostic and statistical manual of mental disorders (DSM-5®). American Psychiatric Pub; 2013 May 22.
2. World Health Organization. Suicide across the world. 2016. Available from: https://www.who.int/mental_health/prevention/suicide/suicideprevent/en/. Accessed 7 January 2019.
3. Office for National Statistics. Suicides in the United Kingdom, 2017 Registration – ONS. 2018. Available at: https://www.ons.gov.uk/releases/suicidesintheuk2017registrations. Accessed 27 Feb. 2019.
4. National Collaborating Centre for Mental Health. Self-harm: Longer Term Management (NICE Clinical Guideline 133). London: National Institute for Clinical Excellence 2011.
5. Hawton, K., Saunders, K.E. and O'Connor, R.C., 2012. Self-harm and suicide in adolescents. *The Lancet*, *379*(9834), pp.2373-2382.
6. McManus S, Bebbington P, Jenkins R et al. Mental health and wellbeing in England: Adult Psychiatric Morbidity Survey 2014. Leeds: NHS Digital. 2016. Available at http://digital.nhs.uk/catalogue/PUB21748. [Accessed 27 Feb. 2019]
7. Sakinofsky I. Repetition of suicidal behaviour. In: Hawton K, and Van Heeringen K eds. The International Handbook of Suicide and Attempted Suicide. Chichester: John Wiley & Sons Ltd, 2000:385–404.
8. Hawton K, Bergen H, Cooper J, et al. Suicide following self-harm: findings from the Multicentre Study of self-harm in England, 2000–2012. Journal of Affective Disorders. 2015 April;(175):147-151.
9. Rodway C, Tham SG, Ibrahim S, et al. Suicide in children and young people in England: a consecutive case series. The Lancet Psychiatry. 2016 Aug 1;3(8):751-9.
10. Jourian TJ. Evolving nature of sexual orientation and gender identity. New Directions for Student Services. 2015 Dec;(152):11-23.
11. King M, Semlyen J, Tai SS, Killaspy H, Osborn D, Popelyuk D, Nazareth I. A systematic review of mental disorder, suicide, and deliberate self harm in lesbian, gay and bisexual people. BMC psychiatry. 2008 Dec;8(1):70.
12. Plöderl M, Wagenmakers EJ, Tremblay P, Ramsay R, Kralovec K, Fartacek C, Fartacek R. Suicide risk and sexual orientation: a critical review. Archives of Sexual Behavior. 2013 Jul 1;42(5):715-27.
13. Hottes TS, Bogaert L, Rhodes AE, et al. Lifetime prevalence of suicide attempts among sexual minority adults by study sampling strategies: a systematic review and meta-analysis. American journal of public health. 2016 May;106(5):e1-2.
14. Haas AP, Eliason M, Mays VM, Mathy RM, Cochran SD, D'Augelli AR, Silverman MM, Fisher PW, Hughes T, Rosario M, Russell ST. Suicide and suicide risk in lesbian, gay, bisexual, and transgender populations: Review and recommendations. Journal of homosexuality. 2010 Dec 30;58(1):10-51.
15. Richards C, Barker M. Sexuality and gender for mental health professionals: A practical guide. Sage; 2013 Oct 15.
16. Whittle S, Turner L, Combs R, et al. Transgender EuroStudy: Legal Survey and Focus on the Transgender Experience of Health Care. Brussels, Belgium: ILGA Europe; 2008. http://tgeu.org/sites/default/files/eurostudy.pdf. Accessed 26 March 2019

For peer review only - http://bmjopen.bmj.com/site/about/guidelines.xhtml

17. Bauer GR, Pyne J, Francino MC, et al. Suicidality among trans people in Ontario: implications for social work and social justice/La suicidabilité parmi les personnes trans en Ontario: implications en travail social et en justice sociale. Serv Soc. 2013;59:35–62.
18. Motmans J, de Biolley I, Debunne S. Being Transgender in Belgium: Mapping the Social and Legal Situation of Transgender People. Brussels: Institute for the Equality of Women and Men; 2010. http://igvmiefh. belgium.be/nl/binaries/34%20-%20Transgender_ENG_tcm336-99783.pdf. Accessed 26 March 2019
19. Grant JM, Mottet LA, Tanis J, et al. Injustice at Every Turn: A Report of the National Transgender Discrimination Survey. Washington, D.C.: National Center for Transgender Equality and National Gay and Lesbian Task Force; 2011. http://www.thetaskforce.org/downloads/reports/reports/ntds_full.pdf. Accessed 26 March 2019.
20. McNeil J, Bailey L, Ellis S, et al. Mental Health Study 2012. Edinburgh: Equality Network; 2012. http://www.scottishtrans.org/ Uploads/Resources/trans_mh_study.pdf. Accessed 26 March 2019.
21. Nock MK, Borges G, Bromet EJ, et al. Cross-national prevalence and risk factors for suicidal ideation, plans and attempts. The British Journal of Psychiatry. 2008 Feb;192(2):98-105.
22. Gratz KL. Risk factors for and functions of deliberate self-harm: An empirical and conceptual review. Clinical Psychology: Science and Practice. 2003 Jun;10(2):192-205.
23. Fliege H, Lee JR, Grimm A, et al. Risk factors and correlates of deliberate self-harm behavior: A systematic review. Journal of psychosomatic research. 2009 Jun 1;66(6):477-93.
24. Gili, M., Castellvi, P., Vives, M., de la Torre-Luque, A., Almenara, J., Blasco, M.J., Cebrià, A.I., Gabilondo, A., Pérez-Ara, M.A., Lagares, C. and Parés-Badell, O., 2018. Mental disorders as risk factors for suicidal behavior in young people: A meta-analysis and systematic review of longitudinal studies. Journal of affective disorders.
25. Hawton K, James A. Suicide and deliberate self harm in young people. BMJ. 2005;330(7496):891–4.
26. Semlyen J, King M, Varney J, et al. Sexual orientation and symptoms of common mental disorder or low wellbeing: combined meta-analysis of 12 UK population health surveys. BMC psychiatry. 2016 Dec;16(1):67.
27. Clements-Nolle K, Marx R, Katz M. Attempted suicide among transgender persons: The influence of gender-based discrimination and victimization. Journal of homosexuality. 2006 Oct 11;51(3):53-69.
28. Meyer IH. Minority stress and mental health in gay men. Journal of health and social behavior.1995 Mar;1:38-56.
29. Meyer IH. Prejudice, social stress, and mental health in lesbian, gay, and bisexual populations: conceptual issues and research evidence. Psychological bulletin. 2003 Sep;129(5):674.
30. Cochran SD, Mays VM. Lifetime prevalence of suicide symptoms and affective disorders among men reporting same-sex sexual partners: results from NHANES III. American journal of public health. 2000 Apr;90(4):573.
31. Bontempo DE, d'Augelli AR. Effects of at-school victimization and sexual orientation on lesbian, gay, or bisexual youths' health risk behavior. Journal of Adolescent Health. 2002 May 1;30(5):364-74.
32. Grossman AH, D'augelli AR, Frank JA. Aspects of psychological resilience among transgender youth. Journal of LGBT Youth. 2011 Mar 29;8(2):103-15.

For peer review only - http://bmjopen.bmj.com/site/about/guidelines.xhtml

33. Brandelli Costa A, Pasley A, Machado WD, et al. The experience of sexual stigma and the increased risk of attempted suicide in young Brazilian people from low socioeconomic group. Frontiers in psychology. 2017 Feb 22;8:192.
34. Kelleher C. Minority stress and health: Implications for lesbian, gay, bisexual, transgender, and questioning (LGBTQ) young people. Counselling psychology quarterly. 2009 Dec 1;22(4):373-9.
35. Taliaferro LA, Muehlenkamp JJ. Nonsuicidal self-injury and suicidality among sexual minority youth: risk factors and protective connectedness factors. Academic pediatrics. 2017 Sep 1;17(7):715-22.
36. Puckett, J.A., Newcomb, M.E., Ryan, D.T., Swann, G., Garofalo, R. and Mustanski, B., 2017. Internalized homophobia and perceived stigma: A validation study of stigma measures in a sample of young men who have sex with men. *Sexuality Research and Social Policy*, *14*(1), pp.1-16.
37. McDermott E, Hughes E, Rawlings V. The social determinants of lesbian, gay, bisexual and transgender youth suicidality in England: a mixed methods study. Journal of Public Health. 2017 Oct 17;40(3):e244-51.
38. Klonsky ED. The functions of deliberate self-injury: A review of the evidence. Clinical psychology review. 2007 Mar 1;27(2):226-39.
39. Skegg K. Self-harm. The Lancet. 2005 Oct 22;366(9495):1471-83.
40. Edmondson AJ, Brennan CA, House AO. Non-suicidal reasons for self-harm: a systematic review of self-reported accounts. Journal of Affective Disorders. 2016 Feb 1;191:109-17.
41. Marshall E, Claes L, Bouman WP, et al. Non-suicidal self-injury and suicidality in trans people: a systematic review of the literature. International review of psychiatry. 2016 Jan 2;28(1):58-69.
42. Miranda-Mendizábal, A., Castellví, P., Parés-Badell, O., Almenara, J., Alonso, I., Blasco, M.J., Cebrià, A., Gabilondo, A., Gili, M., Lagares, C. and Piqueras, J.A., 2017. Sexual orientation and suicidal behaviour in adolescents and young adults: systematic review and meta-analysis. *The British Journal of Psychiatry*, *211*(2), pp.77-87.
43. Marshal MP, Dietz LJ, Friedman MS, et al. Suicidality and depression disparities between sexual minority and heterosexual youth: A meta-analytic review. Journal of adolescent health. 2011 Aug 1;49(2):115-23.
44. E Mann G, Taylor A, Wren B, et al. Review of the literature on self-injurious thoughts and behaviours in gender-diverse children and young people in the United Kingdom. Clinical child psychology and psychiatry. 2018:1359104518812724.
45. Shamseer L, Moher D, Clarke M, et al. Preferred reporting items for systematic review and meta-analysis protocols (PRISMA-P) 2015: elaboration and explanation. Bmj. 2015 Jan 2;349:g7647.
46. Wells G, Shea B, O'Connell D, et al. Newcastle-Ottawa quality assessment scale cohort studies. 2011. http://www.ohri.ca/programs/clinical_epidemiology/oxford. asp.
47. Herzog R, Álvarez-Pasquin MJ, Díaz C, et al. Are healthcare workers' intentions to vaccinate related to their knowledge, beliefs and attitudes? A systematic review. BMC public health. 2013 Dec;13(1):154.
48. Arcelus J, Claes L, Witcomb GL, et al. Risk factors for non-suicidal self-injury among trans youth. The Journal of Sexual Medicine. 2016 Mar 1;13(3):402-12.

For peer review only - http://bmjopen.bmj.com/site/about/guidelines.xhtml

49. Beck AT, Steer RA, Ranieri WF. Scale for suicide ideation: psychometric properties of a self-report version. Journal of clinical psychology. 1988 Jul;44(4):499-505.
50. Bostrom AG, Cronbach J. Development of a clinical instrument to estimate suicide risk. Am J Psychiatry. 1985 Jun 6;142(6):680-6.
51. Muehlenkamp JJ, Gutierrez PM, Osman A, et al. Validation of the Positive and Negative Suicide Ideation (PANSI) Inventory in a diverse sample of young adults. Journal of Clinical Psychology. 2005 Apr;61(4):431-45.
52. Posner K, Brown GK, Stanley B, et al. The Columbia–Suicide Severity Rating Scale: initial validity and internal consistency findings from three multisite studies with adolescents and adults. American Journal of Psychiatry. 2011 Dec;168(12):1266-77.
53. Sansone RA, Wiederman MW, Sansone LA. The self-harm inventory (SHI): Development of a scale for identifying self-destructive behaviors and borderline personality disorder. Journal of clinical psychology. 1998 Nov;54(7):973-83.
54. Nock MK, Holmberg EB, Photos VI, Michel BD. Self-Injurious Thoughts and Behaviors Interview: Development, reliability, and validity in an adolescent sample.
55. Taylor PJ, Dhingra K, Dickson JM, et al. Psychological correlates of self-harm within gay, lesbian and bisexual UK University students. Archives of suicide research. 2018 Sep 28:1-6.
56. Knipe D, Williams AJ, Hannan-Swain S, et al. Psychiatric morbidity and suicidal behaviour in low and middle-income countries: A systematic review and meta-analysis. (*in press*)
57. Harbord RM, Egger M, Sterne JA. A modified test for small-study effects in meta-analyses of controlled trials with binary endpoints. Statistics in medicine. 2006 Oct 30;25(20):3443-57.
58. Popay J, Roberts H, Sowden A, et al. Guidance on the conduct of narrative synthesis in systematic reviews. A product from the ESRC methods programme Version. 2006 Apr 1;1:b92.
59. Deeks JJ, Higgins JP, Altman DG. Analysing data and undertaking meta-analyses. Cochrane handbook for systematic reviews of interventions: Cochrane book series. 2008 Sep 26:243-96.

For peer review only - http://bmjopen.bmj.com/site/about/guidelines.xhtml

PRISMA-P 2015 Checklist

This checklist has been adapted for use with protocol submissions to *Systematic Reviews* from Table 3 in Moher D et al: Preferred reporting items for systematic review and meta-analysis protocols (PRISMA-P) 2015 statement. *Systematic Reviews* 2015 **4**:1

Section/topic	#	Checklist item	Information reported Yes	Information reported No	Line number(s)
**ADMINISTRATIVE INFORMATION**					
**Title**					
Identification	1a	Identify the report as a protocol of a systematic review	√	☐	1
Update	1b	If the protocol is for an update of a previous systematic review, identify as such	☐	√	
**Registration**	2	If registered, provide the name of the registry (e.g., PROSPERO) and registration number in the Abstract	√	☐	48
**Authors**					
Contact	3a	Provide name, institutional affiliation, and e-mail address of all protocol authors; provide physical mailing address of corresponding author	√	☐	4-20
Contributions	3b	Describe contributions of protocol authors and identify the guarantor of the review	√	☐	262-265
**Amendments**	4	If the protocol represents an amendment of a previously completed or published protocol, identify as such and list changes; otherwise, state plan for documenting important protocol amendments	☐	√	
**Support**					
Sources	5a	Indicate sources of financial or other support for the review	√	☐	267-269
Sponsor	5b	Provide name for the review funder and/or sponsor	√	☐	268
Role of sponsor/funder	5c	Describe roles of funder(s), sponsor(s), and/or institution(s), if any, in developing the protocol	√	☐	269
**INTRODUCTION**					
**Rationale**	6	Describe the rationale for the review in the context of what is already known	√	☐	60-112
**Objectives**	7	Provide an explicit statement of the question(s) the review will address with reference to participants, interventions, comparators, and outcomes (PICO)	√	☐	113-118

BioMed Central The Open Access Publisher

Section/topic	#	Checklist item	Information reported: Yes	Information reported: No	Line number(s)
**METHODS**					
**Eligibility criteria**	8	Specify the study characteristics (e.g., PICO, study design, setting, time frame) and report characteristics (e.g., years considered, language, publication status) to be used as criteria for eligibility for the review	√	☐	128-163
**Information sources**	9	Describe all intended information sources (e.g., electronic databases, contact with study authors, trial registers, or other grey literature sources) with planned dates of coverage	√	☐	165-174
**Search strategy**	10	Present draft of search strategy to be used for at least one electronic database, including planned limits, such that it could be repeated	√	☐	Sup file 2
STUDY RECORDS					
Data management	11a	Describe the mechanism(s) that will be used to manage records and data throughout the review	√	☐	181-203
Selection process	11b	State the process that will be used for selecting studies (e.g., two independent reviewers) through each phase of the review (i.e., screening, eligibility, and inclusion in meta-analysis)	√	☐	176-180
Data collection process	11c	Describe planned method of extracting data from reports (e.g., piloting forms, done independently, in duplicate), any processes for obtaining and confirming data from investigators	√	☐	181-203
**Data items**	12	List and define all variables for which data will be sought (e.g., PICO items, funding sources), any pre-planned data assumptions and simplifications	√	☐	197-203
**Outcomes and prioritization**	13	List and define all outcomes for which data will be sought, including prioritization of main and additional outcomes, with rationale	√	☐	148-162
**Risk of bias in individual studies**	14	Describe anticipated methods for assessing risk of bias of individual studies, including whether this will be done at the outcome or study level, or both; state how this information will be used in data synthesis	√	☐	217-226
DATA					
**Synthesis**	15a	Describe criteria under which study data will be quantitatively synthesized	√	☐	210-216
	15b	If data are appropriate for quantitative synthesis, describe planned summary measures, methods of handling data, and methods of combining data from studies, including any planned exploration of consistency (e.g., I^2, Kendall's tau)	√	☐	217-226
	15c	Describe any proposed additional analyses (e.g., sensitivity or subgroup analyses, meta-regression)	√	☐	227-232
	15d	If quantitative synthesis is not appropriate, describe the type of summary planned	√	☐	210-216

BioMed Central
The Open Access Publisher

For peer review only

Section/topic	#	Checklist item	Information reported		Line number(s)
			Yes	No	
**Meta-bias(es)**	16	Specify any planned assessment of meta-bias(es) (e.g., publication bias across studies, selective reporting within studies)	√	☐	217-226
**Confidence in cumulative evidence**	17	Describe how the strength of the body of evidence will be assessed (e.g., GRADE)	√	☐	233-236

For peer review only

BioMed Central The Open Access Publisher

SUPPLEMENTARY FILE 2

Search strategy terms:

(self-harm OR self harm* OR self-injur* OR "self injur*" OR self-cut* OR self-destruct* OR "self destruct*" OR "nonsuicidal self-injur*" OR "non-suicidal self injur*" OR "deliberate self harm" OR "deliberate self-harm" OR DSH OR "self-mutil*" OR overdos* OR self-inflicted injur* OR "self inflicted injur*" OR suicid* OR "parasuicid*" OR para-suicid* OR parasuicid* OR suicidal behav* OR suicide* OR "life-threatening behavio*" OR "suicide ideat*" OR "suicide attempt*" OR "attempted suicide*" OR NSSI)

AND

(moderat* OR mediat* OR "risk facto*" OR mechan* OR predict* OR pathway OR interact* OR "protective facto*" OR facto* OR influence OR correlate* OR precurs* OR "causal facto*")

AND

(transgender* OR transsexual* OR "gender nonconforming" OR "gender identity disorder" OR "gender dysphoria" OR "gender minority" OR lesbian*OR gay* OR bisexual* OR "sexual minority" OR "same-sex" OR homosexual* OR "homosexuality, male" OR "homosexuality, female" OR "gender identity" OR non-heterosexual* OR "non heterosexual*"OR homosexuality OR queer* OR questioning OR "non-binary" OR "non binary" OR "LGBT*" OR "sexual dissident*" OR "sexual and gender minorities" OR "gender variant" OR gender-variant OR genderqueer OR intersex OR "minority groups" OR "TGNC" OR "transgender and gender nonconforming")

AND

(Child* OR adolesc* OR "young people" OR kid* OR pupils OR youth OR juvenile OR "young adult*" OR "young person" OR minor*)

For peer review only
